# RpoS contributes in a host-dependent manner to *Salmonella* colonization of the leaf apoplast during plant disease

**DOI:** 10.3389/fmicb.2022.999183

**Published:** 2022-11-08

**Authors:** Amelia H. Lovelace, Hsiao-Chun Chen, Sangwook Lee, Ziad Soufi, Pedro Bota, Gail M. Preston, Brian H. Kvitko

**Affiliations:** ^1^The Sainsbury Laboratory, Norwich Research Park, Norwich, United Kingdom; ^2^Department of Plant Pathology, University of Georgia, Athens, GA, United States; ^3^Department of Microbiology, University of Georgia, Athens, GA, United States; ^4^Department of Plant Sciences, University of Oxford, Oxford, United Kingdom; ^5^The Plant Center, University of Georgia, Athens, GA, United States

**Keywords:** *Salmonella enterica*, produce-borne illness, *Pseudomonas syringae*, rpoS, apoplast, hydrogen peroxide, biomultiplier

## Abstract

Contaminated fresh produce has been routinely linked to outbreaks of Salmonellosis. Multiple studies have identified *Salmonella enterica* factors associated with successful colonization of diverse plant niches and tissues. It has also been well documented that *S. enterica* can benefit from the conditions generated during plant disease by host-compatible plant pathogens. In this study, we compared the capacity of two common *S. enterica* research strains, 14028s and LT2 (strain DM10000) to opportunistically colonize the leaf apoplast of two model plant hosts *Arabidopsis thaliana* and *Nicotiana benthamiana* during disease. While *S. enterica* 14028s benefited from co-colonization with plant-pathogenic *Pseudomonas syringae* in both plant hosts, *S. enterica* LT2 was unable to benefit from *Pto* co-colonization in *N. benthamiana*. Counterintuitively, LT2 grew more rapidly in *ex planta N. benthamiana* apoplastic wash fluid with a distinctly pronounced biphasic growth curve in comparison with 14028s. Using allelic exchange, we demonstrated that both the *N. benthamiana* infection-depedent colonization and apoplastic wash fluid growth phenotypes of LT2 were associated with mutations in the *S. enterica rpoS* stress-response sigma factor gene. Mutations of *S. enterica rpoS* have been previously shown to decrease tolerance to oxidative stress and alter metabolic regulation. We identified *rpoS-*dependent alterations in the utilization of L-malic acid, an abundant carbon source in *N. benthamiana* apoplastic wash fluid. We also present data consistent with higher relative basal reactive oxygen species (ROS) in *N. benthamiana* leaves than in *A. thaliana* leaves. The differences in basal ROS may explain the host-dependent disease co-colonization defect of the *rpoS*-mutated LT2 strain. Our results indicate that the conducive environment generated by pathogen modulation of the apoplast niche can vary from hosts to host even with a common disease-compatible pathogen.

## Introduction

Fresh produce has become an increasing source for foodborne illnesses over the last several decades. In the United States, fresh produce associated foodborne outbreaks increased from 0.7% of reported outbreaks in the 1970s to 33% in 2011 (Bennett et al., 2014). From 2004 to 2015, fresh produce was the leading cause of food borne illness outbreaks (Fischer and Plunkett, 2015). *Salmonella enterica* is a common causal agent of foodborne illnesses and a significant number of cases of Salmonellosis have been linked to consumption of fresh or minimally processed produce (Hanning et al., 2009; Berger et al., 2010; Kozak et al., 2013; Bennett et al., 2015; Crowe et al., 2015; Fischer and Plunkett, 2015; Carstens et al., 2019). Plant-associated Salmonellosis outbreaks, while infrequent, are both economically disruptive and a recurrent public health concern (Bennett et al., 2015; Crowe et al., 2015). In response to the impacts of plant-associated Salmonellosis, multiple studies have sought to characterize the abiotic and biotic factors that contribute to *S. enterica* colonization of both edible and non-edible portions of crop plants as well as model plant hosts (Cooley et al., 2003; Barak et al., 2005, 2009, 2011; Noel et al., 2010; Meng et al., 2013; Garcia et al., 2014; Potnis et al., 2014, 2015; de Moraes et al., 2017, 2018; Jacob and Melotto, 2019; Montano et al., 2020). Mutational and comparative genetic studies have identified factors involved in *S. enterica* plant attachment, internalization, persistence, and translocation between inoculated to non-inoculated portions of plant hosts (recently reviewed by Zarkani and Schikora, 2021). Based on these studies, different plant hosts and plant host niches often pose distinct fitness requirements for *S. enterica* colonization. These studies have also shown that *S. enterica* colonization of plants is not limited to external plant surfaces. *S. enterica* can become internalized through roots and is able to actively bypass stomata to colonize the leaf apoplastic space (Gu et al., 2011; Karmakar et al., 2018; Rodrigues Oblessuc et al., 2019; Chahar et al., 2021).

The majority of genetic studies that have identified *S. enterica* plant colonization factors have focused on binary plant host-*S. enterica* interactions. However, interactions between *S. enterica* and plants are also modulated by plant-associated microbes (Poza-Carrion et al., 2013). It has been well documented that the activities of bacterial plant pathogens can allow *S. enterica* to more effectively colonize various plant niches (Wells and Butterfield, 1997; Meng et al., 2013; Pollard et al., 2014; Potnis et al., 2014, 2015; Deblais et al., 2021; Cowles et al., 2022). Plant pathogens can act as biomultipliers for *S. enterica*, creating favorable conditions that allow *S enterica* to reach higher loads in plant tissues than they can achieve independently (Barak and Schroeder, 2012). For instance, the plant soft rot necrotrophic pathogen *Pectobacterium carotovorum* secretes plant cell wall degrading enzymes that disrupt plant tissues and increase nutritional availability to *S. enterica* (Cox et al., 2013; George et al., 2016). George et al. (2018) used a TnSeq strategy to identify *S. enterica* 14028s genetic factors contributing to successful co-colonization of *P. carotovorum* tomato soft rot. However, for hemibiotrophic plant pathogens like *Pseudomonas syringae*, that translocate type III secretion systems effectors (T3Es) to subvert plant immunity and cause disease, *S. enterica* may also benefit from other forms of pathogen-associated niche modulation (Barak and Schroeder, 2012).

Plants have a distributed innate immune system structured around two interconnected tiers of immune receptors, Nucleotide-binding domain Leucine-rich Repeat receptors (NLRs) and Pattern Recognition Receptors (PRRs; Ngou et al., 2022). NLRs detect specific plant pathogen effector proteins delivered into the plant cytosol and activate plant immunity in response to plant pathogens. Conversely, Pattern Recognition Receptors (PRRs) are presented on the plant cell surface and are able to bind to cognate conserved “non-self” microbial molecular patterns common to both plant pathogens and non-plant pathogens. The archetypal PRR is *Arabidopsis thaliana* FLS2, which binds to broadly conserved epitopes of bacterial flagellin forming a kinase signaling complex to activate plant immune signaling responses (Zipfel et al., 2004; Chinchilla et al., 2006). It has been demonstrated that *S. enterica* activates PRR-mediated immune responses in diverse plants (Meng et al., 2013; Garcia et al., 2014; Jacob and Melotto, 2019). PRR-mediated immunity results in non-permissive conditions in the apoplast that are widely suppressive to bacterial proliferation (Velasquez et al., 2022). PRR-compromised *A. thaliana* displayed dysbiosis resulting in elevated populations of non-plant-pathogenic bacterial endophytes (Xin et al., 2016; Chen et al., 2020). For a given plant host, disease-compatible *P. syringae* translocate T3Es that collectively suppress PRR-mediated immune signaling, either evade or suppress signaling by host NLRs, and increase water and nutrient availability in the apoplast (Xin et al., 2018; Lovelace and Ma, 2022). *S. enterica* has been previously observed to benefit from apoplastic water soaking caused by hemibiotrophic *Xanthomonas* plant pathogens (Potnis et al., 2015). From a sociomicrobiology framework, the plant pathogen-modulated conducive apoplastic niche provides “common goods” allowing other bacteria co-occupying the apoplast to hitch a free ride and derive a reproductive benefit (Macho et al., 2007; Rufian et al., 2018; Chen et al., 2020). However, we still lack a detailed understanding of the metabolic and stressor landscape that distinguishes the conducive niche generated by plant pathogens from the suppressive non-permissive apoplastic niche, as well as to what degree plant pathogen-modulated apoplastic niches vary from host-to-host or pathogen-to-pathogen.

*Salmonella enterica* subsp. *enterica* Typhimurium strains, 14028s and LT2 have been workhorse strains for the characterization of *S. enterica*, genetics, physiology, and host interactions and have been used routinely in studies of *S. enterica* plant colonization. In this study, we compared the competency of 14028s and LT2 (strain DM10000), to benefit from co-colonization of the leaf apoplastic space with the plant pathogen partner *P. syringae* pv. *tomato* DC3000 (*Pto*) in two divergent model plant hosts, *A. thaliana* and *Nicotiana benthamiana*. *Pto* is a well characterized model plant pathogen that is natively disease compatible with *A. thaliana* Col-0 and is also disease compatible with the common laboratory accession of *N. benthamiana* when the effector gene *hopQ* is deactivated to prevent immune recognition by the Roq1 NLR receptor (Wei et al., 2007; Yao et al., 2013; Schultink et al., 2017). We determined that LT2 did not derive a benefit from co-colonization of the *N. benthamiana* leaf apoplast with *Pto* and linked that phenotype to a newly identified and presumed null allele of the *rpoS* sigma factor. We also determined that there are statistically elevated basal ROS levels in *N. benthamiana* leaves compared to *Arabidopsis* leaves. The differences in basal ROS may explain the host-dependent *Pto* infection-associated colonization phenotype of the *rpoS*-mutated LT2 strain. In addition to identification of *rpoS* as a genetic factor contributing to *S. enterica* disease co-colonization in a host-dependent manner, our work is consistent with observations that the disease-associated conducive apoplastic niche can vary from host to host and pose distinct fitness requirements (Helmann et al., 2019).

## Materials and methods

### Plant growth and bacterial culturing conditions

*Arabidopsis thaliana* Col-0 seeds suspended in sterile 0.1% agarose were sown in SunGrow Professional potting mix and stratified in darkness for 1 day at 4°C before being grown in a growth chamber (Conviron A1000) with 14-h light (70 μmol) at 23°C. Plants were removed from the chamber at 4 weeks and kept at 12-h day and 12-h night conditions in the growth room prior to inoculation (4–5 weeks old) or apoplastic extractions (6–7 weeks). *N. benthamiana* were sown in the same potting mix amended with 1 g L^−1^ Peter’s 20–20-20 fertilizer and grown in a growth chamber with 12 h day at 26°C (125 μmol) and 12 h night at 23°C. Two weeks after sowing, seedlings were transplanted into 6-inch pots and fertilized. Plants were removed from the chamber at 5 weeks and kept in the growth room prior to inoculations or apoplastic extractions (6–9 weeks old). Leaves from 4-week-old *N. benthamiana* plants were used for apoplastic fluid isolation. *P. syringae* pv. *tomato* strain DC3000 (*Pto*) strains, and *S. enterica* serovar Typhimurium strains used in this study are listed in Supplementary Table S1. All *Pto* strains were grown on King’s B (KB) medium (per 1 l = 20.0 g proteose peptone 3, 0.4 g MgSO_4_·7H_2_O, glycerol 10 ml, 2.0 g K_2_HPO_4_·3H_2_O, with 18 g agar for solidified media) with 60 μg mL^−1^ of rifampicin at 30°C. *S. enterica* strains were grown routinely in Lysogeny Broth (LB) medium (per 1 l = 10 g tryptone, 5 g yeast extract, 10 g NaCl, pH 7.5 + with 15 g agar for solidified media) at 37°C. To enumerate *S. enterica* populations from a mixed population, samples were grown at 42°C.

### Plant inoculation and sampling procedures

To prepare *S. enterica* bacterial inocula, overnight cultures made from single colonies were incubated in LB at 37°C, pelleted using centrifugation, suspended in 0.25 mm MgCl_2_, and were diluted to an optical density at 600 nm (OD_600_) of 0.8 (approximately 5 × 10^8^ CFU mL^−1^), as determined using a Biospectrometer (Eppendorf, Hamburg, Germany). *Pto* inoculum was prepared as described in Lovelace et al. (2018) and diluted to OD_600_ = 0.8. Bacterial inocula for both individual and co-inoculations were further diluted in 0.25 mm MgCl_2_ to the desired concentrations. *S. enterica* strains at 5 × 10^5^ CFU mL^−1^ (approximately LOG 3.5 CFU/cm^2^)were mixed with *Pto* or *Pto*Δ*hrcC* (a Type III Secretion System mutant) to a final *Pto* concentration of 5 × 10^6^ CFU mL^−1^ (approximately LOG 4.5 CFU/cm^2^) and syringe-inoculated into four *A. thaliana* leaves per plant. *S. enterica* strains at 5 × 10^5^ CFU mL^−1^, were mixed with *Pto*Δ*hopQ* (disease compatible with *N. benthamiana*) or *Pto*Δ*hrcC* to a final concentration of 5 × 10^5^ CFU mL^−1^ and syringe inoculated into fully expanded *N. benthamiana* leaves. All inocula were serial diluted and plated on appropriate media with antibiotics to verify bacterial concentrations. All inoculated hosts were incubated for 3 days under high humidity (90–100% RH) at ambient room temperature. At 3 days post inoculation, four 4 mm diameter leaf discs (∼0.5 cm^2^ total) per treatment were collected with a 4 mm diameter biopsy punch. Leaf discs were macerated for 2 min in 0.1 ml of 0.25 mm MgCl_2_ using a SpeedMill Plus homogenizer (Analytik Jena, Jena, Germany) and the bacterial CFU/cm^2^ leaf tissue was determined by serial dilution spot plating as described in Liu et al. (2015). Initial bacterial populations (CFU/cm^2^ leaf tissue) from co-inoculations were determined from independent plant samples (Supplementary Figure S1). All plant inoculation assays were repeated three times with 3 plants per treatment. For co-inoculation assays, statistical differences between mean final bacterial populations were determined using a 2- tailed *t*-test for each strain (*p* < 0.05).

### Extraction of apoplastic wash fluid and GC–MS analysis

Apoplastic wash fluid (AWF) was crude extracted using vacuum infiltration as described by O'Leary et al. (2014) with slight modifications. Whole *A. thaliana* plants or fully expanded *N. benthamiana* leaves were cut and placed into a 500 ml beaker with 300 ml of sterile distilled water. Repeated cycles of vacuum at 95 kPa for 2 min followed by slow release of pressure were applied until leaves were fully infiltrated. Excess water was blotted from plant tissue before leaves were rolled into 20 ml syringes which were placed into 50 ml conical tubes. Tubes were centrifuged at 1,000 rpm for 10 min at 4°C and the fractions were pooled and stored at −80°C. AWF samples were filter sterilized using 0.2 μM RapidFlow filters for subsequent experiments.

For metabolomic analyses, *N. benthamiana* apoplastic wash fluid (NbAWF) samples were collected as described by O'Leary et al. (2016). NbAWF samples were prepared for GC–MS analysis using a modified version of the method of Lisec et al. (2006). One hundred and fifty microliter samples of NbAWF were mixed with 700 μl of methanol, supplemented with 10 μg mL^−1^ of ribitol, and then shaken at 70°C for 10 min, followed by centrifugation for 5 min at 11,000× *g*. Next, 700 μl of supernatant was removed and mixed sequentially by vortexing with 375 μl of cold chloroform and 500 μl of cold ddH_2_O. Samples were centrifuged at 2,200× *g* for 15 min, then 250 μl of supernatant was transferred to a fresh tube and dried in a vacuum concentrator without heat. The samples were derivatized in 29 μl of pyridine containing 20 mg mL^−1^ methoxyamine and 50 μl of N-Methyl-N-(trimethylsilyl) trifluoroaceamide (MSTFA) as described previously (Lisec et al., 2006). GC–MS analysis was performed as described previously (O'Leary et al., 2016). Area values of identified BAWF compounds from six biological replicates were averaged and normalized against a ribitol internal standard and standard deviation was calculated for each compound.

### Bacterial growth assays

Bacterial inocula were generated as described above for all strains. Cell suspensions in 0.25 mm MgCl_2_ were standardized to an OD_600_ of 0.8 (5 × 10^8^ CFU mL^−1^). Aliquots of 300 μl of bacterial inocula were diluted into 2.7 ml of NbAWF, AtAWF or M9 glucose medium (CSHL, 2010) to a final concentration of approximately 5 × 10^7^ CFU mL^−1^. To make macronutrient and micronutrient amended NbAWF, 1,000 × concentrated macronutrients and micronutrients including sodium chloride, magnesium sulfate, ammonium sulfate, calcium chloride, glucose, potassium phosphate, potassium chloride, and sodium phosphate, were dissolved in distilled water and filter sterilized using 0.2 μM filters before being diluted to 1× in NbAWF. The same volume of water was used as a control. Aliquots of 400 μl were inoculated into 5 replicate wells of a Bioscreen honeycomb plate (Bioscreen Technologies, Bertinoro, Italy). The OD_600_ was measured every hour for up to 36 h in a Bioscreen C plate reader with low to medium shaking at 22°C (Bioscreen Technologies, Bertinoro, Italy). Raw absorbance readings were normalized by subtracting the initial absorbance readings from subsequent hourly readings.

For the competition assay, standardized inocula of each *S. enterica* strain and *Pto* were diluted in combination with either 0.25 mm MgCl_2_ or an inoculum partner for individual or co-inoculations, respectively, in M9 glucose medium or NbAWF to a final concentration of 5 × 10^6^ CFU mL^−1^ of each bacterium. Aliquots of 400 μl were inoculated into 5 replicate wells of a Bioscreen honeycomb plate and the growth (measured as OD_600_) was monitored until peak OD_600_ was achieved; 1 day for samples grown in NbAWF and 2 days for samples grown in minimal media. Serial dilutions were plated on appropriate plates with antibiotics to determine the initial and final population sizes of both *S. enterica* strains and *Pto* measured as CFU mL^−1^. Initial populations were measured from three aliquots of the original suspension and final populations were measured from individual wells in the Bioscreen honeycomb plate. Statistical differences between mean initial and final bacterial populations under single and co-inoculating conditions were determined using a 2-way ANOVA for each strain (*p* < 0.05). At least two independent experiments were performed for all growth assays.

### Gene fragment swap by allelic exchange

*Salmonella enterica* knock-out clones were generated in the LT2 strain background using the pR6KT2G suicide vector which allows for SacB-mediated sucrose counter-selection using methods defined by Stice et al. (2020) with modifications. The promoter and gene sequence of *rpoS* (STM14_3526) from 14028s was obtained from KEGG Gene using the organism code “seo.” Flanks of 300 bp preceding and following the first 352 bp of *rpoS* were synthesized with *attB1* and *attB2* extensions for Gateway clonase recombination as double stranded DNA gblocks by Twist Bioscience (Supplementary Table S1). The synthesized gene fragment was cloned with BP clonase II into pR6KT2G according to the manufacturer’s recommendations (Thermo Scientific, Waltham, MA, United States). The cleaned reaction mixture was electroporated into competent *E. coli* MaH1 *pir*^+^ cells and transformed cells were grown on LB amended with 10 μg mL^−1^ gentamicin. Allelic replacement constructs were confirmed by BsrGI digest and sequencing before transformed into electrocompetent *E. coli* RHO5 *pir*^+^ cells.

The wild-type LT2 strain and RHO5 pR6KT2G::*rpoS* donor strain were mated on LB plates amended with 250 μg mL^−1^ diaminopimelic Acid (DAP). Single crossover merodiploids were recovered from the mating mixture on LB plates amended with 10 μg mL^−1^ gentamicin. Two merodiploid colonies were selected for counter selection in a liquid culture of 1 ml LB and 3 ml 1 M sucrose for 24 h at 37°C. Following counter selection, a portion of the diluted mixture was plated on LB plates amended with X-gluc (5-bromo-4-chloro-3-indolyl-beta-D-glucuronic acid, cyclohexylammonium salt). Candidate allelic replacement strains were not blue in color indicating eviction of the plasmid construct. Genomic DNA extractions were performed on candidate colonies using the Gentra Puregene kit according to the manufacturer’s instructions (Qiagen, Hilden, Germany). Candidate colonies were screened using external primers (Supplementary Table S1) by PCR using Phusion HiFi polymerase according to the manufacturer’s instructions (New England BioLabs, Ipswich, MA, United States). Candidates with the expected PCR fragment size were sequenced using external primers to confirm the gene deletion.

The resulting LT2∆*rpoS*^1-352^ strain was replaced with the *rpoS* 352 bp gene fragment from 14028s using the same homologous recombination procedure used to generate the mutants. Flanks of 300 bp preceding and following the 14028s *rpoS* gene fragment were synthesized with *attB1* and *attB2* extensions for Gateway compatibility as double stranded DNA gblocks by Twist Bioscience (Supplementary Table S1). This gene fragment was cloned as described above and candidate knock-in strains were screened using external primers by PCR; the resulting gene fragment was digested using the AvaII restriction enzyme according to the manufacturer’s instructions to distinguish 14028s and LT2 genotypes (New England BioLabs, Ipswich, MA, United States). Candidates with the expected PCR and digest fragment sizes were sequenced using the external primers to confirm the 14028s *rpoS* gene knock-in clones.

### Hydrogen peroxide tolerance

LT2 and LT2 *rpoS*_14028s_ strains were tested for viable bacterial population and growth in response to treatment with different concentrations of hydrogen peroxide. From overnight cultures prepared as described above, strains were pelleted by centrifugation and washed twice with fresh LB and adjusted to OD_600_ of 0.8 (approximately 5 × 10^7^ CFU mL^−1^). Aliquots of 200 μl bacterial inocula were treated with 0, 4, and 8 mm hydrogen peroxide in clear flat bottom plates on an orbital shaker at 1500 rpm at room temperature for 2 h. An equal volume of LB was used as media control. Serial dilutions were plated on LB plates to determine the population sizes of both *S. enterica* strains at the end point as CFU mL^−1^ relative to the media control. Populations were measured from three aliquots of the original suspension and final populations were measured from individual wells. For measuring bacterial growth, plates were set up as described above and OD_600_ was measured using the SpectraMax iD3 reader (Molecular Devices, San Jose, CA, U.S.A) with the kinetic mode for 24 h at 22°C. Two independent experiments were performed for CFU counts and growth curves.

### Carbon source utilization

Sugars, sugar alcohols, and organic carboxylic acids identified in the NbAWF GC–MS profile were selected as carbon sources for testing of differential utilization between *S. enterica* LT2 and LT2 *rpoS*_14028s_ strains. Lactose was used as a negative control carbon source not utilized by *S. enterica*. Cultures of each *S. enterica* strain were generated from a single colony in LB at 37°C as described above. Cultures were pelleted by centrifugation and washed twice with fresh 1X M9 salt. The bacterial concentration was adjusted to OD_600_ of 0.8 and was diluted 1 to 10 (approximately 5 × 10^6^ CFU mL^−1^). To prepare the 10 X concentration stocks, 4% of carbon sources including sucrose, D-glucose, galactose, D-fructose, L-malic acid, succinic acid, myo-inositol, and lactose were dissolved in distilled water and filter sterilized using 0.2 μM filters before dilution to 0.4% of each carbon source as well as a water only control. Aliquots of 200 μl of inoculum with carbon source were inoculated into 4 replicates well at a 96-well flat bottom microplate with medium shaking at 22°C in Biotek Synergy II microplate reader (Winooski, VT). OD_600_ was measured every hour for up to 30 h and raw absorbance readings were normalized by subtracting the initial value of absorbance.

### DAB and POX assays

*Pto* and derivatives were inoculated in *A. thaliana* and *N. benthamiana* to examine the *in situ* accumulation of hydrogen peroxide. *Pto* inocula were prepared as described above. For *N. benthamiana*, 1 × 10^6^ CFU mL^−1^ of *Pto*Δ*hopQ* (compatible with *N. benthamiana*) or *Pto*Δ*hrcC* were syringe-inoculated into fully expanded leaves. For *A. thaliana*, concentrations of 1 × 10^7^ CFU mL^−1^ *Pto* or *Pto*Δ*hrcC* were used. 0.25 mm MgCl_2_ was treated as buffer control in both host plants. All inoculated plants were incubated under high humidity (90–100% RH). 6 h and 24 h post inoculation, four 4 mm leaf discs were collected for 3,3′-diaminobenzidine (DAB) staining. Leaf discs were collected in a 12-well microplate and submerged in DAB staining buffer as described in Daudi and O'Brien (2012). In short, leaf discs were gently vacuum infiltrated at 95 kPa for 2 min, then the plate was covered with aluminum foil and placed on a standard laboratory shaker for 4 h at 80–100 rpm at room temperature. Following, the foil was removed, and DAB staining buffer was replaced with the bleaching buffer (ethanol: acetic acid: glycerol = 3:1:1). The plate was placed in a boiling water bath (~90–95°C) for 5–10 min until the chlorophyll had been removed. Leaf discs were taken out and excessive liquid was blotted off. Color intensity of brown precipitate was quantified using ImageJ (Schneider et al., 2012), 1/3 of the area from edges were omitted to exclude the wounding-associated signal from the cut edges of the discs. For each experiment, four plants were pooled as biological replicates and experiments were performed three times.

For the peroxidase (POX) assay, 4 mm-size of leaf discs from *A. thaliana* and *N. benthamiana* were collected and washed in 1× Murashige–Skoog solution (Murashige and Skoog, 1962) on a rotary shaker for 1 h to remove the signal from damage. *Pto* or *PtoΔhrcC* were used as inocula for *A. thaliana*, and *Pto*Δ*hopQ* and *PtoΔhrcC* were used in *N. benthamiana.* Leaf discs were then individually placed in a 96-well clear flat bottom plate with 50 μl of 5 x 10^7^CFU mL^−1^ bacterial suspension in 1× Murashige–Skoog solution and gently vacuum infiltrated for 2 min at 95 kPa. Leaf discs were submerged with bacterial inocula and incubated at 80–100 rpm shaking at room temperature for 20 h after the vacuum infiltration. After the incubation, leaf discs were removed from individual well and POX activity was measured as described in Mott et al., 2018 (Mott et al., 2018). Briefly, 50 μl of 1 mg mL^−1^ 5-aminosalicylic acid solution (pH 6.0) with 0.01% hydrogen peroxide was pipetted to each well and set for 2 min. 20 μl of 2 m NaOH solution was then added to each well to stop the reaction. Quantification of POX was measured at OD_600_ immediately.

## Results

### *Salmonella enterica* LT2 has a host-dependent defect in co-colonization of the *Nicotiana benthamiana* apoplast during *Pseudomonas syringae* disease

*Salmonella enterica* 14028s and LT2 strain DM10000 were assessed for their capacity to achieve increased loads in the leaf apoplast of *A. thaliana* and *N. benthamiana* when co-inoculated with a common host-compatible *P. syringae* (*Pto*) pathogen partner. Suspensions of *S. enterica* were standardized to 5 × 10^5^ CFU mL^−1^ and pressure inoculated directly into the leaf apoplast using a needless syringe. *S. enterica* strains were either inoculated singly or co-inoculated with host-compatible *Pto* or *Pto*Δ*hopQ* at inoculum concentrations used routinely in each plant host (5 × 10^6^ CFU mL^−1^ for *A. thaliana* and 5 × 10^5^ CFU mL^−1^ for *N. benthamiana* respectively), or the same concentrations of the *Pto*Δ*hrcC* control strain, which lacks a functional Type III Secretion System (T3SS) and is incapable of translocating T3Es and causing disease. Three days post inoculation, the bacterial loads of *S. enterica* and *Pto* strains in leaves were determined by leaf tissue maceration and dilution plating under appropriate selective conditions.

**Figure 1:**
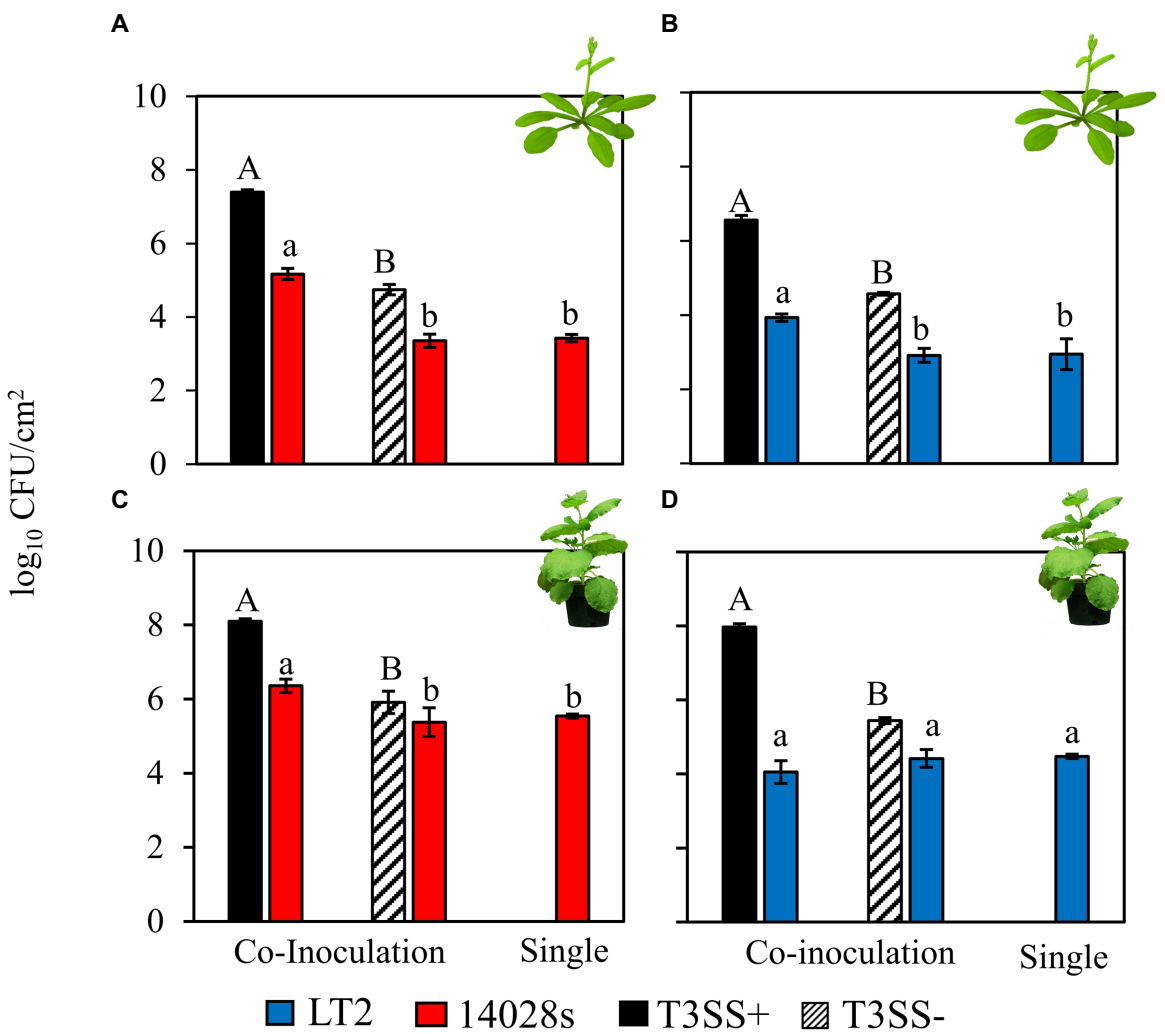
*Salmonella enterica* LT2 has a host-dependent defect in co-colonization of the *Nicotiana benthamiana* apoplast during *P. syringae* disease. Bacterial populations of *S. enterica* strains **(A,C)** 14028s (red), and **(B,D)** LT2 (blue) with co-inoculation partner *P. syringae* (*Pto*) or *Pto*∆*hopQ* with a functional Type III Secretion System (T3SS+, black) and *Pto*∆*hrcC* without a functional T3SS (T3SS−, striped) Inocula were syringe infiltrated into model plant hosts, **(A,B)** *A. thaliana* Col-0 at a concentration of 5 × 10^6^ CFU mL^−1^ for *Pto* strains and 5 × 10^5^ CFU mL^−1^ for *S. enterica* strains and **(C,D)** *N. benthamiana* at a concentration of 5 × 10^5^ CFU mL^−1^ for all strains. Bacterial populations were measured as log colony forming units per cm^2^ of leaf tissue (log_10_ CFU/cm^2^) 3 days post-inoculation. Data are means ± SD (*n* = 3 plants). Different letters indicate significant differences (2-tailed *t*-test for each strain at *p* < 0.05). Three independent experiments were conducted with similar results.

Both *S. enterica* 14028s and LT2 benefitted from infection co-colonization in the *A. thaliana* apoplast based on their increased in load versus either independent *S. enterica* inoculation or co-inoculation with the disarmed, non-pathogenic, *Pto*Δ*hrcC* T3SS− control strain (Figures 1A,B). Without the pathogenic *Pto* strain the *S. enterica* populations decreased slightly compared with the day 0 population (Supplementary Figure S1). This indicates that T3SS-dependent *Pto* infection resulted in conducive conditions in the *A. thaliana* apoplast that could be opportunistically exploited by both *S enterica* strains. *S. enterica* 14028s also benefited from *Pto*Δ*hopQ* infection co-colonization in *N. benthamiana*, as has been observed previously (Figure 1C; Meng et al., 2013). However, although *S. enterica* populations had increased by day 3 compared with the day 0 population in each treatment (Supplementary Figure S1), *S. enterica* LT2 achieved similar populations in *N. benthamiana* when co-inoculated with pathogenic *Pto*Δ*hopQ*, non-pathogenic *PtoΔhrcC* and when inoculated independently (Figure 1D). As only this combination of plant host and *S. enterica* strain resulted in *Pto* infection-independent colonization, this implicated both *N. benthamiana* plant host and LT2 strain factors in the phenotype.

### The *Salmonella enterica* LT2 *Pto* infection-independent colonization is unlikely to be caused by nutritional competition with *Pto*

**Figure 2:**
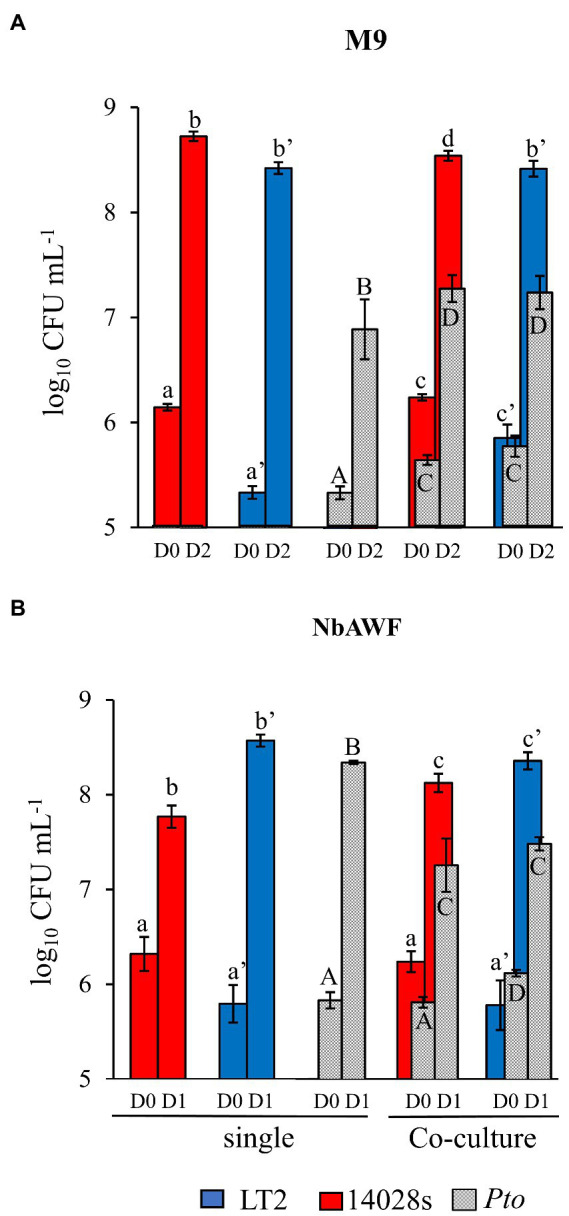
*Salmonella enterica* is competent for competition with *P. syringae* during co-culture in *ex planta N. benthamiana* apoplastic wash fluid. Bacterial populations of *P. syringae* (*Pto*, grey) and *S. enterica* strains LT2 (blue) and 14028s (red) after inoculation in **(A)** M9 glucose medium (M9) and **(B)** *N. benthamiana* apoplastic wash fluid (NbAWF). Bacterial populations were measured as log colony forming units per mL of culture (log_10_ CFU mL^−1^) on day 0, 1, and 2. Data are means ± SD (*n* = 3–5). Different letters indicate significant differences (2-way ANOVA for each strain at *p* < 0.05). Asterisk indicates significant difference between co-inoculation partners (2 tailed *t*-test at *p* < 0.05). Two independent experiments were conducted with similar results.

Based on the assumption that, as a specialist plant pathogen, *Pto* would be better equipped to utilize nutrients available in the *N. benthamiana* apoplast than *S. enterica*, our initial hypothesis was that the LT2 *Pto* infection-independent colonization might result from *Pto* outcompeting *S. enterica* LT2 for a growth-limiting nutritional resource. To address this, we recovered *N. benthamiana* apoplastic wash fluid (NbAWF), using standard methods for apoplastic wash fluid extraction. We determined endpoint populations *via* dilution plating of the *S. enterica* strains and *Pto* both individually and while co-cultured in filter-sterilized *ex planta* NbAWF or M9 glucose medium as a control. Both *S. enterica* strains grew well in NbAWF fluid and M9 glucose medium both individually and when co-cultured with *Pto* (Figure 2). In fact, counter to our initial hypothesis, it was the *Pto* partner that reached lower endpoint population sizes in NbAWF when co-cultured with either *S. enterica* strain than when cultured individually (Figure 2B). This experiment did not support *S. enterica*-*Pto* nutritional competition as being a major mediator of LT2 *Pto* infection-independent colonization in *N. benthamiana*.

### *Salmonella enterica* LT2, counter-intuitively, grows more rapidly in *Nicotiana benthamiana* AWF than *Salmonella enterica* 14028s and with a distinct biphasic growth curve

**Figure 3:**
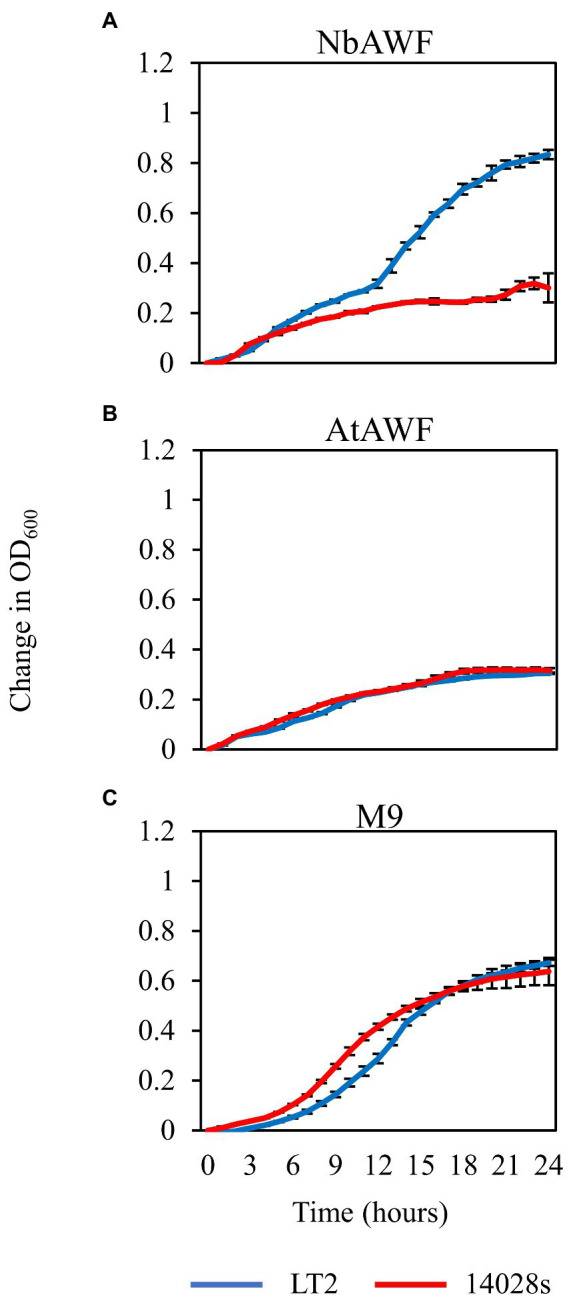
*Salmonella enterica* strain LT2 exhibits more rapid growth with a pronounced biphasic growth pattern in *ex planta N. benthamiana* apoplastic wash fluid. Growth curves of *S. enterica* LT2 (blue) and *S. enterica* 14028s (red) in **(A)** *N. benthamiana* apoplastic wash fluid (NbAWF), **(B)** *A. thaliana* apoplastic wash fluid (AtAWF), and **(C)** M9 minimal media. Cultures were incubated at 22°C, and the OD_600_ was recorded every hour. Growth was measured as the mean change in OD_600_. Error bars show standard deviation (*n* = 5 wells). Two independent experiments were conducted with similar results.

While culturing *S. enterica* in *ex planta* NbAWF we made the unexpected observation that LT2 grew more rapidly than 14028s and with a more pronounced biphasic growth curve (Figure 3A). Conversely, both *S. enterica* strains displayed largely similar growth behavior in filter-sterilized *A. thaliana* AWF and M9 glucose medium (Figures 3B,C). The biphasic growth of LT2 in NbAWF seemed consistent with diauxic growth associated with catabolite repression. Based on this hypothesis, we were curious whether biphasic growth in NbAWF could be nutritionally suppressed. We augmented NbAWF with components of M9 glucose medium in which both *S. enterica* strains grew similarly. The biphasic growth of LT2 in NbAWF was not suppressed by augmentation with *s*odium chloride, magnesium sulfate, ammonium sulfate, or calcium chloride (Supplementary Figure S2). However, augmentation with either glucose or potassium phosphate suppressed NbAWF biphasic growth with LT2 growth being more starkly suppressed than 14028s by these two compounds (Supplementary Figure S3). Compared to augmentation with glucose, augmentation with potassium phosphate had a temporary effect on biphasic growth suppression. To test whether the phosphate anion or potassium cation was responsible for transient suppression of biphasic growth we supplemented NbAWF with potassium chloride, potassium phosphate, or sodium phosphate. In both cases where a phosphate anion was used, *S. enterica* biphasic growth was transiently suppressed while augmentation with potassium chloride resulted in similar biphasic growth to that of the water control (Supplementary Figure S4). The observed growth suppression *via* nutrient augmentation is consistent with variation in the regulation of catabolite repression between the two strains.

### Both the *Pto* infection-independent colonization and NbAWF biphasic growth phenotypes of LT2 are associated with mutations in the *rpoS* gene

**Figure 4:**
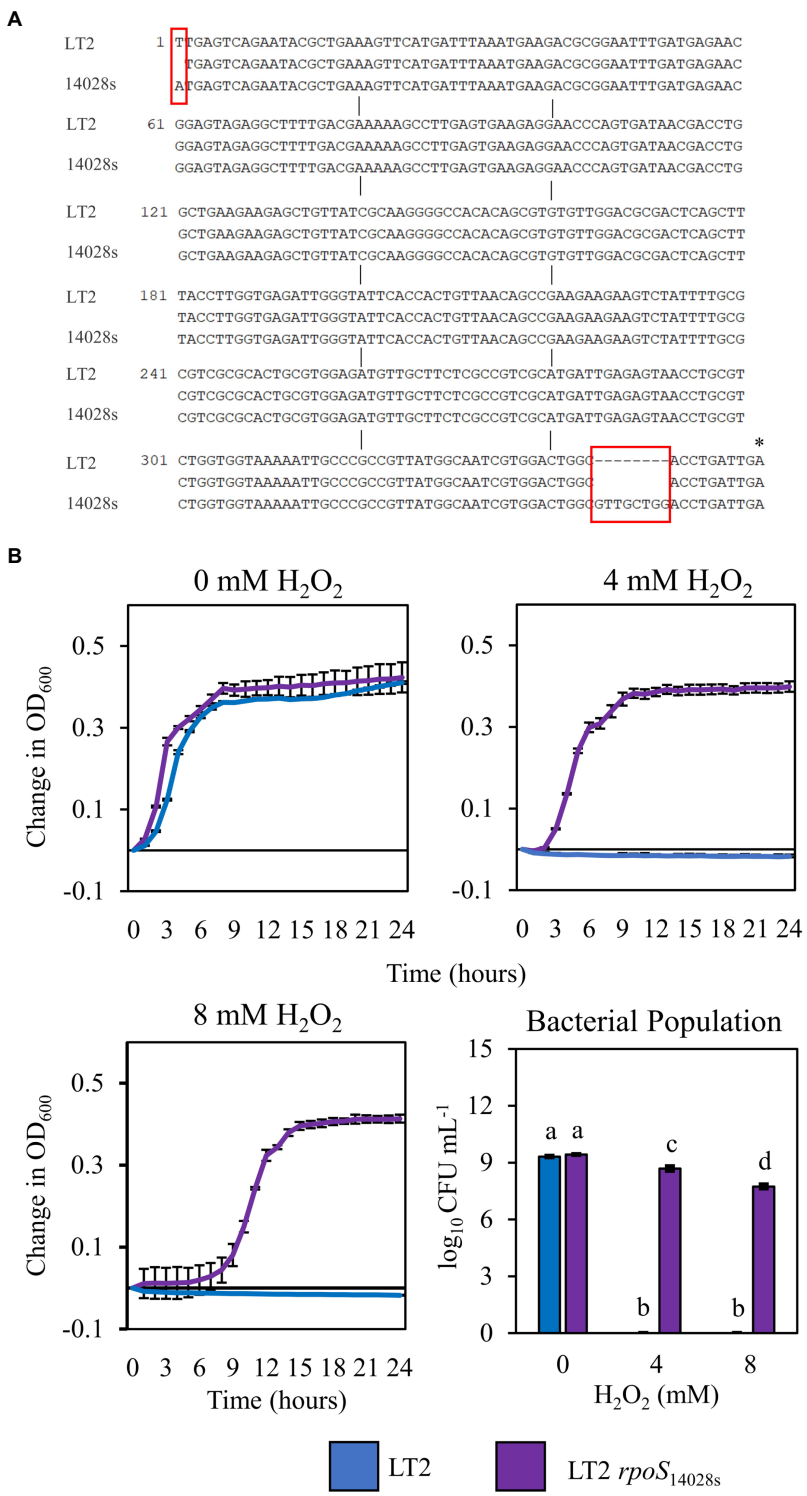
Restoration of the *rpoS* ORF of *S. enterica* LT2 increases tolerance to H_2_O_2_. **(A)** Nucleotide sequence alignment of the first 362 bp of the *rpoS* gene in *S. enterica* strains LT2 and 14028s. Red boxes indicate nucleotide differences between strains. Asterisk indicates premature stop codon. **(B)** Growth curves of *S. enterica* LT2 (blue) and LT2 *rpoS*_14028s_ (purple) in LB with 0, 4, 8 mm H_2_O_2_ amended. Cultures were aliquoted into 4 replicate wells, incubated at 22°C, and the OD_600_ was recorded every hour. Growth was measured as the average change in measured OD_600_ with standard deviation error bars (*n* = 4 wells). Bacterial populations were measured as log colony forming units per mL after 2 h treatment with H_2_O_2_. Data are means ± SD (*n* = 3). Different letters indicate significant differences (2-way ANOVA for each strain at *p* < 0.05). Two independent experiments were conducted with similar results.

**Figure 5:**
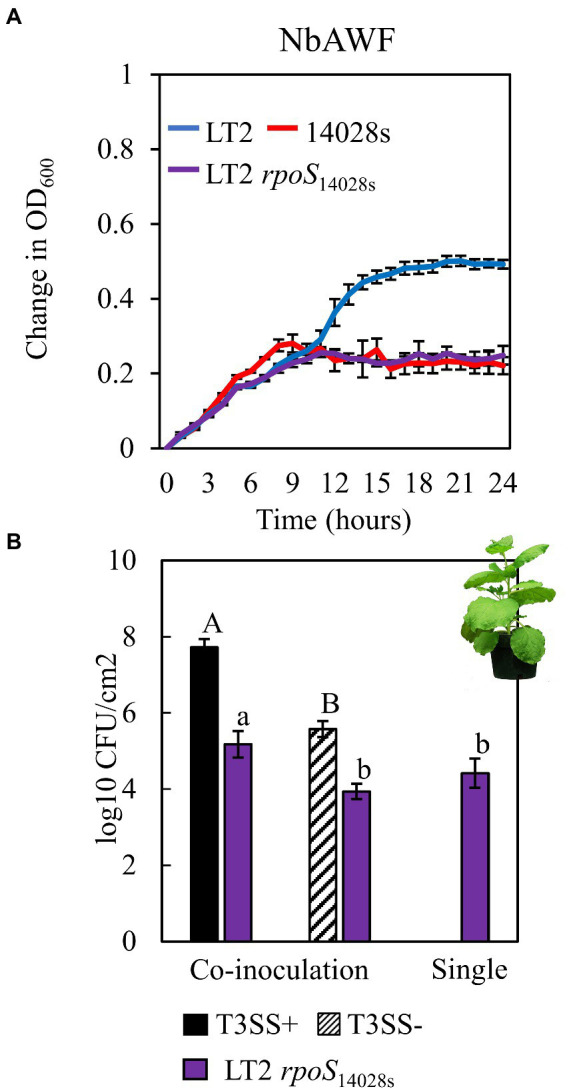
Restoration of the *rpoS* ORF in *S. enterica* LT2alters both *ex planta* and *in planta* phenotypes. **(A)** Growth curves of LT2 (blue), 14028s (red), and LT2 *rpoS*_14028s_ (purple) in *N. benthamiana* apoplastic wash fluid (NbAWF). Cultures were aliquoted into 5 replicate wells, incubated at 22°C, and the OD_600_ was recorded every hour. Growth was measured as the average change in measured OD_600_ with standard deviation error bars (*n* = 5 wells). Two independent experiments were conducted with similar results. **(B)** Population of *S. enterica* LT2 *rpoS*_14028s_ in *N. benthamiana,* co-inoculated with *P. syringae* (*Pto*) with (T3SS+, black) and without (T3SS−, striped) a functional Type III Secretion System. *Pto*∆*hopQ* and *Pto*∆*hrcC were* used as T3SS+ and T3SS− respectively. Inocula were syringe-infiltrated at concentrations defined in Figure 1. Data are means ± SD (*n* = 3 plants). Bacterial populations were measured as log colony forming units per cm^2^ of leaf tissue (log_10_ CFU/cm^2^) 3 days post-inoculation. Data are means ± SD (*n* = 3 plants). Different letters indicate significant differences (2-tailed *t*-test for each strain at *p* < 0.05). Three independent experiments were conducted with similar results.

The infection-independent colonization phenotype of LT2 in *N. benthamiana* implicated both *S. enterica* strain and *N. benthamiana* host factors. A notable genetic difference between the LT2 and 14028s is the mutation of the LT2 *rpoS* sigma factor AUG start codon to the less-efficient UUG start codon (Swords et al., 1997; Wilmes-Riesenberg et al., 1997). This mutation results in lower RpoS protein levels and has been shown to be associated with reduced stress tolerance (Wilmes-Riesenberg et al., 1997; Jarvik et al., 2010). While confirming the *rpoS* sequences of 14028s and LT2, we identified an additional 8-bp deletion mutation starting at nucleotide position 344 in the *rpoS* gene of LT2 DM10000. This 8-bp indel introduces a premature UGA stop codon at *rpoS*_LT2_ nucleotide position 352 resulting in a predicted 117 aa truncated protein versus the 330 aa residue full-length *rpoS* ORF of 14028s and a likely null allele (Figure 4A). To our knowledge this 117 aa truncated allele has not been described previously and is not common among other LT2 isolates. We were curious whether these *rpoS* mutations contributed to our observed LT2 disease co-colonization defect and NbAWF growth phenotypes. We used two-step allelic exchange to delete the LT2 *rpoS* 1–352 region and subsequently replaced the deletion with the corresponding *rpoS*_14028s_ coding sequence resulting in *rpoS* with an ATG start codon and the full-length open reading frame. Consistent with LT2 *rpoS*_14028s_ having restored function compared to the native truncated *rpoS*_LT2-117_ allele, we observed that LT2 *rpoS*_14028s_ gained a dramatically improved tolerance to H_2_O_2_ (Figure 4B). When we tested the growth of the LT2 *rpoS*_14028s_ strain in NbAWF, we observed that it had lost the pronounced biphasic growth phenotype and displayed growth behavior similar to 14028s (Figure 5A). In addition, we observed that LT2 *rpoS*_14028s_ gained the capacity to benefit from *Pto* disease co-colonization in *N. benthamiana* (Figure 5B).

### RpoS restoration alters utilization of L-malic acid, an abundant carbon source in *Nicotiana benthamiana* apoplastic wash fluid

**Figure 6:**
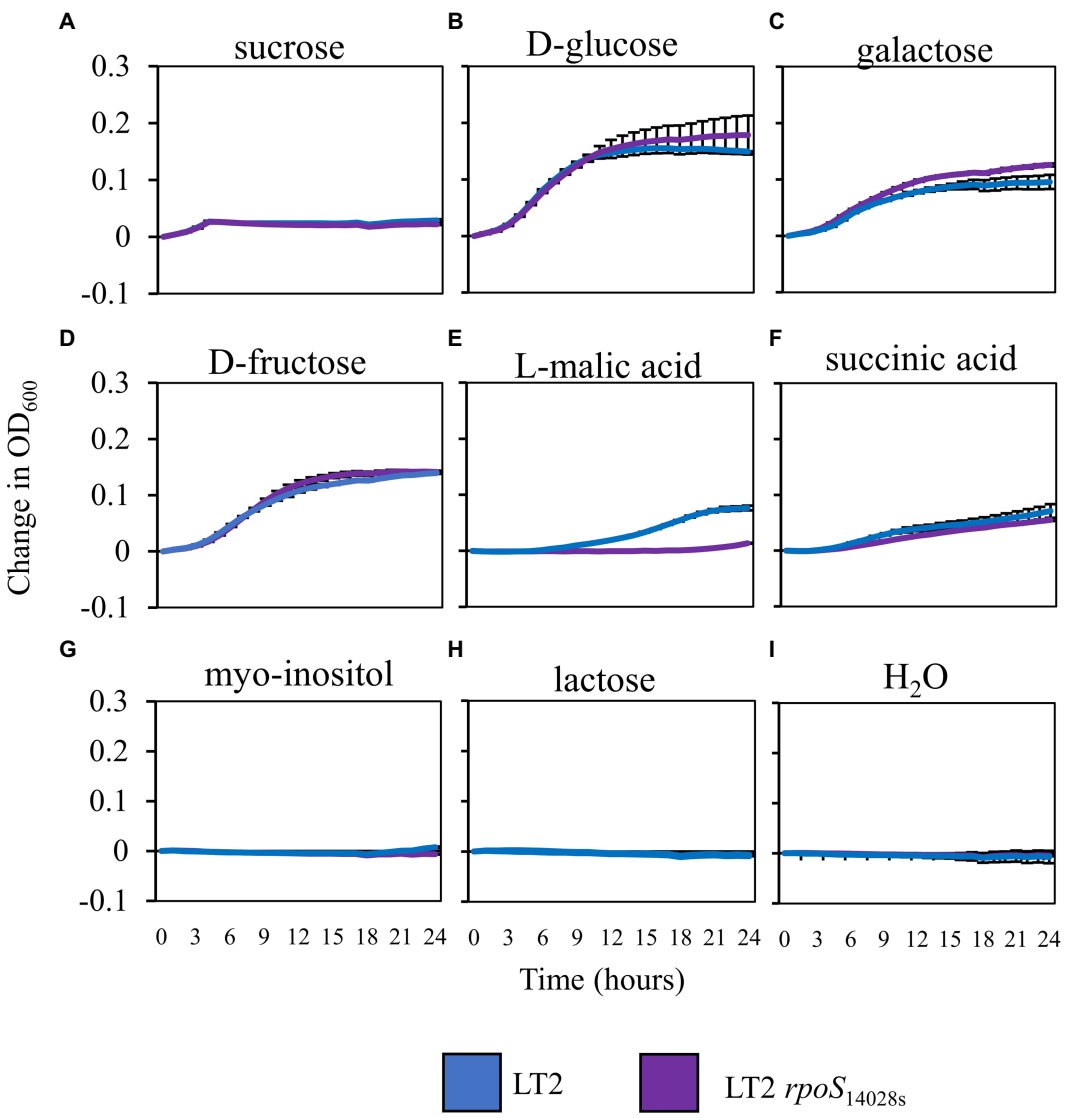
*Salmonella enterica* growth in M9 with NbAWF carbon sources. Growth curves of *S. enterica* LT2 (blue) and LT2 *rpoS*_14028s_(purple) in M9 medium supplemented with different carbon sources LT2 (blue) and LT2 *rpoS*_14028s_(purple) in M9 medium **(A)** sucrose, **(B)** D-glucose, **(C)** galactose, **(D)** D-fructose, **(E)** L-malic acid, **(F)** succinic acid, **(G)** myo-inositol, **(H)** lactose, and mock control **(I)** H_2_O. Cultures were aliquoted into 4 replicate wells, incubated at 22°C, and the OD_600_ was recorded hourly. Growth was measured as the average change in measured OD_600_ with standard deviation error bars (*n* = 4 wells). Two independent experiments were conducted with similar results.

Based on the previously observed capacity of glucose to suppress LT2 biphasic growth in NbAWF, we speculated that the enhanced biphasic growth of LT2 compared with 14028s and LT2 *rpoS*_14028s_ in *ex planta* NbAWF could be caused by *rpoS-*dependent differential utilization patterns for a carbon source found in NbAWF. Analysis by GC–MS of NbAWF detected 69 compounds including sugars and sugar derivatives, amino acids and amino acid derivatives, and organic acids (Supplementary Table S2). We tested LT2 and LT2 *rpoS*_14028s_ for differential utilization of seven carbon sources identified in NbAWF (fructose, glucose, sucrose, galactose, myo-inositol, malic acid, and succinic acid) as the sole carbon sources in M9 medium. We also tested lactose as a negative control which was not detected in NbAWF and is not utilized by *S. enterica*. Of these compounds, there was a notable difference in the growth behavior of the two strains in M9 L-malic acid medium with LT2 showing a decreased lag phase compared with LT2 *rpoS*_14028s_ (Figure 6). Of note, malic acid was identified as the apoplastic metabolite with the second highest relative concentration in NbAWF after sucrose (Supplementary Table S2). Thus, the *rpoS-*associated variations in biphasic growth in NbAWF may be mediated by variation in the utilization malic acid present in NbAWF.

### Evidence for higher basal ROS in *Nicotiana benthamiana* compared to *Arabidopsis thaliana* leaves

**Figure 7:**
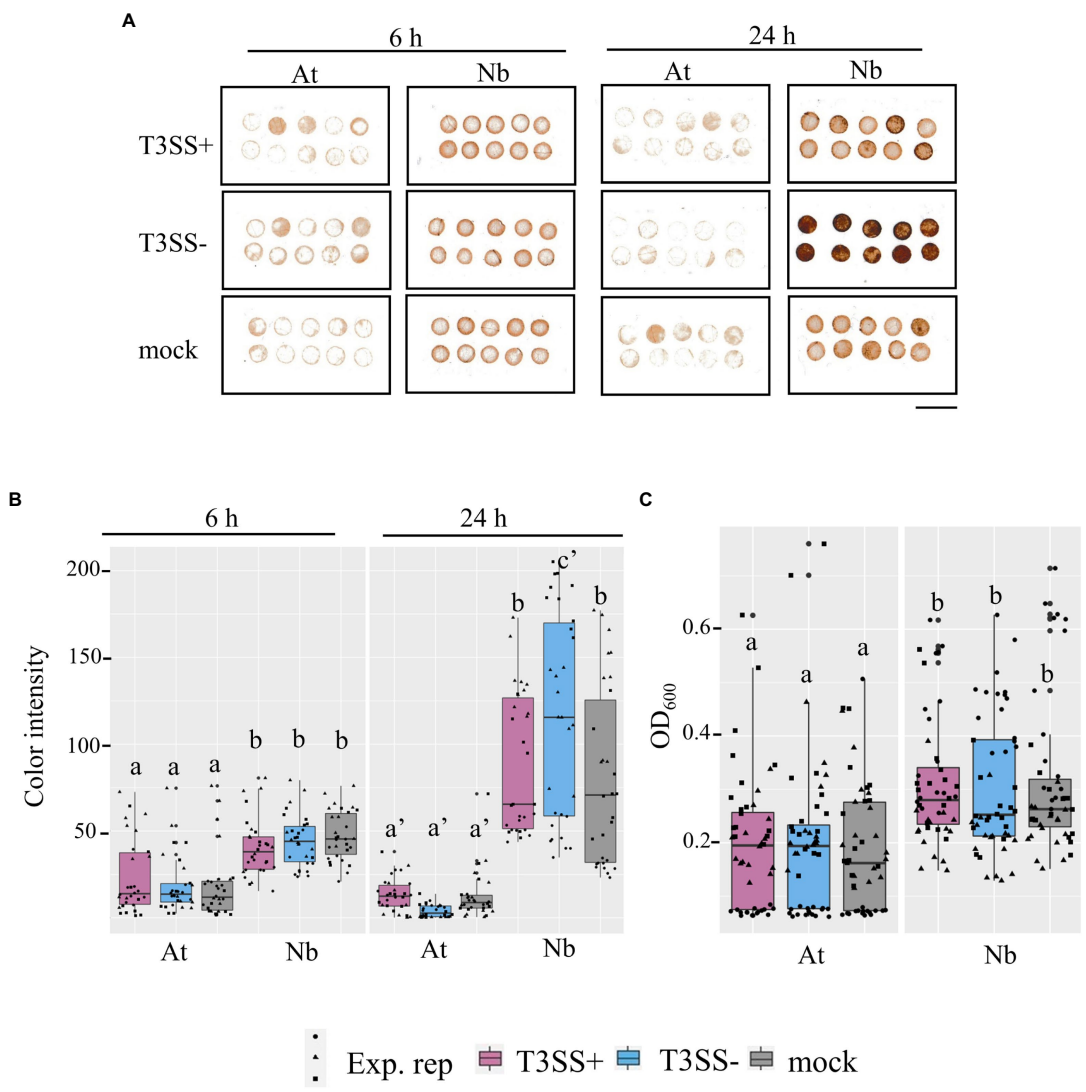
Evidence for higher basal ROS in *N. benthamiana* levels compared to *A. thaliana* leaves. **(A,B)** *in situ* H_2_O_2_ in *N. benthamiana* and *A. thaliana* inoculated with *P. syringae* (*Pto*) with (T3SS+) and without (T3SS−) a functional Type III Secretion System. The detected H_2_O_2_ level within inoculated tissues of *N. benthamiana* (Nb) and *A. thaliana* (At) were measured with DAB staining at both an earlier time point (6 h) and later time point (24 h). T3SS+ strains, *Pto* and *Pto∆hopQ,* were used in *A. thaliana* and *N. benthamiana*, respectively. *Pto∆hrcC* was used as T3SS− treatment for both plants. Whole leaves were syringe-infiltrated with bacterial suspension (1 × 10^7^ CFU mL^−1^ for *A. thaliana* and 1 × 10^6^ CFU mL^−1^ for *N. benthamiana*) or 0.25 mm MgCl_2_ treatment as mock treatment. 4 mm diameter of leaf discs were collected at each time point post inoculation. Scale bar, 10 mm. **(C)** Peroxidase (POX) assay shows peroxidase levels in *N. benthamiana* than in *A. thaliana*. Leaf disks from Col-0 (At) and *N*. *benthamiana* (Nb) were vacuum infiltrated with bacterial suspension (5 × 10^7^ CFU mL^−1^) and incubated for 20 h. T3SS+ and T3SS− strains used for both plants were described as above. Absorbance of OD_600_ was measured as POX activity. Graphs show data from three experimental replicates (Exp. rep) with means ± SE, *n* = 16 discs from 4 plants. Different letters indicate statistically significant differences as analyzed by 1-way ANOVA (Tukey test, *p* < 0.05).

Reactive oxygen species play important roles in plant immune signaling (Shapiguzov et al., 2012; Qi et al., 2017). Based on the *rpoS*-association for infection co-colonization in *N. benthamiana* but not *A. thaliana,* and the stark *rpoS*-dependent H_2_O_2_ tolerance phenotype of LT2, we were curious whether there might be differences in the ROS concentrations of *N. benthamiana* leaves compared with *A. thaliana* leaves. We used two distinct assays to quantify ROS in leaf discs of both hosts. 1) Diaminobenzidine (DAB) staining in which H_2_O_2_-mediated oxidation of DAB results in the production of a brown precipitate in leaf tissue that can be quantified by image analysis and 2) the peroxidase (POX) assay in which the H_2_O_2_-mediated oxidation of 5-aminosalyclic acid by apoplastic plant peroxidases results in a color change that is measured spectrophotometrically. We used these techniques to monitor ROS levels after treatment with disease-compatible *Pto* pathogen (T3SS+) or non-pathogenic *Pto*Δ*hrcC* (T3SS−) compared with a MgCl_2_ buffer control. Although, the 24 h treatment with the *N. benthamiana* disease-compatible *Pto*Δ*hopQ* resulted in less ROS signal than treatment with the *Pto*Δ*hrcC* strain based on the DAB assay, ROS signal was significantly higher in *N benthamiana* than *A. thaliana* in every treatment and time point tested (Figure 7). This is consistent with *N. benthamiana* maintaining higher relative basal ROS than *A. thaliana*. It is possible that the elevated basal ROS of *N. benthamiana* combined with the reduced ROS tolerance of LT2 might directly contribute to the LT2 *Pto* infection-independent colonization phenotype.

## Discussion

In this study, we observed that *S. enterica* subsp. *enterica* Typhimurium LT2 strain DM10000 is unable to benefit from co-colonization of the *N. benthamiana* apoplast with a disease-compatible *P. syringae* plant pathogen partner. We demonstrated that the *N. benthamiana* LT2 *Pto* infection-independent colonization phenotype was linked to a presumed null allele of the *rpoS* sigma factor. The *rpoS*_LT2-117_ mutation was also associated with increased sensitivity to hydrogen peroxide, pronounced biphasic growth *in ex planta* NbAWF, and altered utilization of L-malic acid. In addition, we present evidence for higher relative levels of basal ROS in *N. benthamiana* than in *A. thaliana* which, when considered in the context of the low hydrogen peroxide tolerance of LT2 strain DM10000, could potentially mediate the LT2 *Pto* infection-independent colonization phenotype.

The host-microbe and microbe-microbe interactions that influence the outcomes of three-way human enteric pathogen, plant host, plant pathogen interactions are complex. Multiple studies have observed that *S. enterica* plant colonization is impacted by plant genotype and that different *S. enterica* factors influence fitness and colonization of different plant hosts (Barak et al., 2011; de Moraes et al., 2017; Jacob and Melotto, 2019; Montano et al., 2020). Highlighting that not all plant pathogen-modulated niches are equally exploitable by *S. enterica*, only a subset of *Xanthomonas* tomato pathogens conferred a co-colonization fitness advantage to *S. enterica* serovars Enteritidis and Baildon (Potnis et al., 2015). Recent work by Cowles et al. (2022) determined that the *Xanthomonas* T3E AvrHah1, associated with leaf water soaking, is both necessary and sufficient for increased persistence of 14028s during *Xanthomonas* disease co-colonization of tomato leaves. Deblais et al. (2021), demonstrated that S*. enterica* LT2 (strain JSG626) persistence on tomato leaves was enhanced by the bacterial tomato pathogen *Clavibacter michiganensis* subsp. *michiganensis* but not by *Xanthomonas gardneri*. Deblais et al. (2021) also noted that LT2 strain JSG626 displayed better persistence on tomato than six other *S. enterica* strains in preliminary testing. It would be interesting to characterize whether these strains also vary in their capacity to benefit from disease co-colonization.

Amongst the set of identified genetic polymorphisms between 14028s and LT2, the LT2 *rpoS* UUG start codon mutation is both well known and well characterized due to its role in reduced stress tolerance and LT2 virulence attenuation (Fang et al., 1992; Swords et al., 1997; Wilmes-Riesenberg et al., 1997). We determined that the replacement of LT2 DM10000 *rpoS* with the 14082s *rpoS* full length coding sequence greatly enhanced tolerance to hydrogen peroxide and allowed LT2 DM10000 to benefit from disease co-colonization in the *N. benthamiana* apoplast. Previous studies have identified both RpoS and oxidative stress tolerance as contributing to *S. enterica* colonization of legume sprouts (Barak et al., 2005; Wahlig et al., 2019). Barak et al. (2005), reported that transposon-inactivation of *rpoS* resulted in decreased attachment by *S. enterica* Newport to alfalfa sprouts based on decreased RpoS-mediated production of curli and cellulose. However, our use of direct leaf apoplastic infiltration likely abrogates attachment requirements as a component of *S. enterica* plant colonization. Wahlig et al. (2019) inferred that an observed decrease in LT2 H_2_O_2_ tolerance also mediated reduced colonization of *Medicago trunculata* sprouts by LT2 but not 14028s in a manner dependent on the functional MtFLS2 flagellin immune receptor. Conversely Zarkani et al. (2019) reported no major difference in the colonization and persistence of 14028s and LT2 on tomato leaves using either direct infiltration or dip inoculation. In addition, in two independent studies by de Moraes et al. (2017) and Montano et al. (2020), transposon insertions in *rpoS* were not identified among *S. enterica* factors contributing to 14028s colonization of either tomato fruit pericarps or lettuce leaves, respectively.

While comparing our observations to previously published work, there are two issues worth noting. First, the majority of comparative and mutational genetic studies that have identified *S. enterica* plant colonization factors have focused on independent *S. enterica* colonization, while co-colonization of the plant pathogen-modulated apoplastic niche very likely poses distinct fitness requirements. Second, in our work we identified a novel and presumed null allele of *rpoS* in LT2 strain DM10000 which may confound direct comparison with published results using *S. enterica* LT2. Considering that *rpoS* mutations can be selected unintentionally during laboratory culture (Notley-McRobb et al., 2002; Ferenci, 2003), it may be beneficial to characterize the *rpoS* integrity of other *S. enterica* strains used in plant-colonization experiments.

To our knowledge, the rpoS_LT2-117_ allele has not been previously reported and is not common among LT2-derived strains. While the LT2 rpoS_UUG_ allele is associated with levels of H_2_O_2_ tolerance comparable to that of a WT *rpoS* allele, null mutations in *rpoS* are associated with a dramatic loss in H_2_O_2_ tolerance and catalase activity (Wilmes-Riesenberg et al., 1997). The rpoS_LT2-117_ allele is presumably also a null mutation based on its H_2_O_2_ sensitivity phenotype and degree of ORF truncation (Figure 4). In addition to regulation of catalase expression and H_2_O_2_ tolerance, the RpoS sigma factor regulates tolerance to acid stress, high osmolarity, high temperature, and promotes survival during extended starvation (Wilmes-Riesenberg et al., 1997). RpoS also plays a major role in the regulation of *S. enterica* metabolic transition into stationary phase (Levi-Meyrueis et al., 2014). Paradoxically, although *rpoS* is upregulated during stationary phase and plays a role the regulation of starvation survival genes, *rpoS* inactivating mutations can also provide a selective advantage under nutrient limitation conditions (Notley-McRobb et al., 2002; Ferenci, 2003). The antagonistic pleiotropy of *rpoS* mutants providing both a selective advantage under nutrient limitation and a selective disadvantage in response to physiological stressors has been studied extensively in *E. coli* but is also supported in *S. enterica* (Notley-McRobb et al., 2002; Ferenci, 2003; Robbe-Saule et al., 2007; Levi-Meyrueis et al., 2014). In *E. coli*, mutations in *rpoS* can be readily recovered by selecting strains for improved growth on a non-preferred carbon source such as succinate in the absence of physiological stressors (Notley-McRobb et al., 2002; Dong et al., 2009). Under these selective regimes *rpoS* alleles are often recovered with reduced activity rather than full null alleles presumably because these alleles provide the strains with limited stress tolerance (Ferenci, 2003). The relevancy of *rpoS* antagonistic pleiotropy for *S. enterica* plant colonization either with or without plant pathogen partners in a natural setting is unclear. Presumably, physiological stressors associated with colonization of plant tissues would provide purifying selection against *rpoS-*inactivating mutations in *S. enterica*, but improved capacity for growth on non-preferred carbon sources common in plant-associated niches might also provide *rpoS* mutants with a selective advantage under certain conditions. Although the LT2 DM10000 rpoS_LT2-117_ allele was presumably unintentionally selected during laboratory culture, *S. enterica* Enteritidis and Typhi clinical isolates with *rpoS-*deactivating mutations and catalase defects have been previously identified indicating that these mutations are not solely an artifact of the laboratory (Robbe-Saule et al., 2003; Betancor et al., 2009).

Our work also indicates that phenotypes based solely on behavior in *ex planta* apoplastic wash fluid should be interpreted cautiously. The pronounced biphasic growth of LT2 DM10000 in *ex planta* NbAWF is dissimilar to the behavior of the strain *in planta* during *N. benthamiana* disease co-colonization. Ideally, NbAWF represents a partial metabolic snapshot of the water extractable fraction of the apoplastic compartment present at the time of sampling. However volatile and/or reactive compounds may not survive processing, filter sterilization, and storage. Apoplastic wash fluid cannot fully capture the dynamic complexity of active microbe-plant biotic interactions. We determined that NbAWF contains a wide diversity of potential carbon and nitrogen sources that *S. enterica* could utilize and serves as an effective growth medium for both 14028s and LT2. The co-occurrence of the *rpoS*-associated LT2 DM10000 *N. benthamiana* infection-independent colonization phenotype and the pronounced *ex planta* NbAWF biphasic growth phenotype seems to be an extraordinary, although artifactual coincidence. Although RpoS in *S. enterica* has been more extensively studied in regards to its role in stress tolerance, over 45% of the RpoS regulon is associated with both the positive and negative regulation of metabolic genes (Levi-Meyrueis et al., 2014). The suppressive effect of glucose or phosphate augmentation on the NbAWF biphasic growth phenotype is consistent with *rpoS*-dependent alteration to diauxic growth utilization patterns of a non-preferred carbon source in NbAWF. Exogenous phosphate has previously been shown to suppress catabolite control of the expression of *csr* genes which regulates motility, carbon storage, and virulence in *S. enterica*, which could explain why we observe suppression of biphasic growth with exogenous phosphate in NbAWF (Teplitski et al., 2006). Although our tests for *rpoS-*dependent altered utilization of NbAWF identified carbon sources was far from exhaustive, we did identify *rpoS-*dependent alteration in the utilization of L-malic acid in M9 medium with decreased growth lag observed in the LT2 strain with the *rpoS*_LT2-117_ allele (Figure 6). Dong et al. (2009) identified that *E. coli* selected for spontaneous *rpoS*-deactivating mutations also had shortened generation times when grown on malate as a sole carbon source. Although the connection between altered utilization of L-malic acid and NbAWF biphasic growth is solely correlative, it is notable that we identified malic acid as the second highest relative concentration metabolite in NbAWF, while Gao et al. (2020) did not identify malic acid among *A. thaliana* apoplastic wash fluid metabolites in which the two strains grew similarly.

The accumulation of apoplastic ROS and H_2_O_2_ is a well established component of the plant immune response (Qi et al., 2017). Consistent with this, we observed statistically elevated DAB staining in *N. benthamiana* at 24 h after treatment with non-pathogenic *Pto*Δ*hrcC.* However, notably we measured statistically higher ROS signal in *N. benthamiana* than in *A. thaliana* with all treatments and timepoints (Figure 7). This could be indicative either of higher ROS production or of lower antioxidant activity in *N. benthamiana* relative to *A. thaliana*. Significant differences in defense responses between cultivars of lettuce, such as reactive oxygen species burst and callose deposition, were found to have a positive correlation with apoplastic persistence of 14028s as well as *E. coli* O157:H7 (Jacob and Melotto, 2019). While disease compatible pathogens can suppress aspects of immune signaling, this suppression is quantitative. For instance, *Pto* collectively requires catalases to detoxify external H_2_O_2_ to cause disease which would presumably be unnecessary if apoplastic H_2_O_2_ were completely suppressed during a compatible disease interaction (Guo et al., 2012). Likewise, the rpos_LT2-117_ -associated hydrogen peroxide sensitivity and catalase deficiency of LT2 DM10000 may be adequate to explain the *N. benthamiana* disease co-colonization defect.

The variation in *Pto* infection-dependent colonization competence of *S. enterica* LT2 in *A. thaliana* or *N. benthamiana* indicates a dissimilarity of the pathogen-modulated apoplastic environments between these two hosts. Apoplastic plant defenses are most typically studied in the context of specialist plant pathogens. However, these specialists, in addition to their active mechanisms to suppress aspects of the plant immune response, are likely well adapted to evade or tolerate intrinsic or immune-associated stressors of the apoplast. Conversely, human enteric pathogens like *S. enterica* may provide a clearer window into the selective pressures of the naïve non-permissive and the pathogen-modulated conducive apoplastic niches. The physiological and transcriptional responses of *Salmonella* spp. and their close relatives to various stressors have been extensively studied and may therefore be more readily interpretable in the context of apoplastic plant defenses (Rychlik and Barrow, 2005; Shen and Fang, 2012). In our work, we identified a plant host-dependent contribution for the global regulatory sigma factor RpoS to infection co-colonization. Characterizing the contributions of *S. enterica* RpoS-regulated genes such as catalases and other oxidative stress tolerance genes would help to clarify the potential role of basal ROS as the mechanistic mediator of the *N. benthamiana Pto* infection-independent colonization phenotype. In addition, previous studies have demonstrated the utility of conducting *in planta* transcriptomics of *S. enterica* (Jacob et al., 2021) and the use of TnSeq to identify genetic factors contributing to *S. enterica* disease co-colonization of tomato during soft rot disease (George et al., 2018). Future work based on *S. enterica* transcriptomics or TnSeq during disease co-colonization with T3SS-dependent plant pathogens could provide powerful new insights into the landscape of the pathogen-modulated conducive apoplastic niches in diverse plant hosts and how plant pathogens may impact *S. enterica* colonization of diverse plants.

## Data availability statement

The original contributions presented in the study are included in the article/Supplementary material, further inquiries can be directed to the corresponding author.

## Author contributions

AL: Conceptualization, methodology, validation, formal analysis, investigation, writing, review and editing, visualization, and funding. H-CC: Conceptualization, methodology, validation, formal analysis, investigation, writing, review and editing, and visualization. SL: Formal analysis and investigation. ZS: Formal analysis, investigation, data curation, and writing. PB: Methodology, resources, and supervision. GP: Conceptualization, review and editing, supervision, project administration, and funding. BK: Conceptualization, resources, writing review and editing, supervision, project administration, and funding. All authors contributed to the article and approved the submitted version.

## Funding

This work was supported by grants from the United States Department of Agriculture: USDA-NIFA 2018-07750 awarded to AL, National Science Foundation IOS 1844861 to BK and University of Georgia College of Agriculture and Environmental Science Interdisciplinary Seed Grant Program: Ensuring Safe Food and Water to BK. ZS received support through a CARA Fellowship.

## Conflict of interest

The authors declare that the research was conducted in the absence of any commercial or financial relationships that could be construed as a potential conflict of interest.

## Publisher’s note

All claims expressed in this article are solely those of the authors and do not necessarily represent those of their affiliated organizations, or those of the publisher, the editors and the reviewers. Any product that may be evaluated in this article, or claim that may be made by its manufacturer, is not guaranteed or endorsed by the publisher.

## Supplementary material

SUPPLEMENTARY FIGURE S1 Starting population of *Salmonella enterica* LT2, 14028s and the co-inoculated *Pseudomonas syringae* partner strains in *Arabidopsis thaliana* and *Nicotiana benthamiana*. Bacterial populations of *S. enterica* strains **(A,C)** 14028s (red), and **(B,D)** LT2 (blue) with co-inoculation partner *P. syringae* (*Pto*) or *Pto*∆*hopQ* with a functional Type III Secretion System (T3SS+, black) and *Pto*∆*hrcC* without a functional T3SS (T3SS−, striped). Inocula were syringe infiltrated into model plant hosts, **(A,B)** *A. thaliana* Col-0 at a concentration of 5 × 10^6^ CFU mL^−1^ for *Pto* strains and 5 × 10^5^ CFU mL^−1^ for *S. enterica* strains and **(C,D)** *N. benthamiana* at a concentration of 5 × 10^5^ CFU mL^−11^ for all strains. Bacterial populations were measured as log colony forming units per cm^2^ of leaf tissue (log_10_ CFU/cm^2^) immediately after the inoculation. Data are means ± SD (*n* = 3 plants). Different letters indicate significant differences (2-tailed t-test for each strain at *p* < 0.05). Three independent experiments were conducted with similar results.

SUPPLEMENTARY FIGURE S2 *Salmonella enterica* biphasic growth in *N. benthamiana* apoplastic wash fluid is unaltered after augmentation with specific macro and micronutrients. Growth curves of *S. enterica* LT2 (blue) and *S. enterica* 14028S (red) in *N. benthamiana* apoplastic wash fluid augmented with **(A)** 10 mM sodium chloride, **(B)** 5 mM magnesium sulfate, **(C)** 10 mM ammonium sulfate, **(D)** 1 mM calcium chloride, and **(E)** water. Cultures were aliquoted into 5 replicate wells, incubated at 22°C, and the OD_600_ was recorded every 2 h. Growth was measured as the average change in measured OD_600_ with standard deviation error bars (*n* = 5 wells). Two independent experiments were conducted with similar results.

SUPPLEMENTARY FIGURE S3 *Salmonella enterica* strain biphasic growth in *N. benthamiana* apoplastic wash fluid is suppressed by glucose and phosphate augmentation. Growth curves of *S. enterica* LT2 (blue) and *S. enterica* 14028s (red) in *N. benthamiana* apoplastic wash fluid augmented with **(A)** 25 mM glucose, **(B)** 10 mM potassium phosphate, and **(C)** water. Cultures were aliquoted into 5 replicate wells, incubated at 22°C, and the OD_600_ was recorded every 2 h. Growth was measured as the average change in OD_600_. Error bars show standard deviation (*n* = 5 wells). Two independent experiments were conducted with similar results.

SUPPLEMENTARY FIGURE S4 *Salmonella enterica* biphasic growth in *N. benthamiana* apoplastic wash fluid is suppressed by the phosphate anion. Growth curves of *S. enterica* LT2 (blue) and *S. enterica* 14028s (red) in *N. benthamiana* apoplastic wash fluid supplemented with **(A)** 10 mM potassium chloride, **(B)** water, **(C)** 10 mM potassium phosphate, and **(D)** 10 mM sodium phosphate. Cultures were aliquoted into 5 replicate wells, incubated at 22°C, and the OD_600_ was recorded every hour. Growth was measured as the average change in measured OD_600_ with standard deviation error bars (*n* = 5 wells). Two independent experiments were conducted with similar results.
