# Supplementary figures and images for: RpoS contributes in a host-dependent manner to Salmonella colonization of the leaf apoplast during plant disease
Source: Front Microbiol. 2022 Nov 8;13:999183. doi: 10.3389/fmicb.2022.999183 (PMC9679226; doi:10.3389/fmicb.2022.999183)

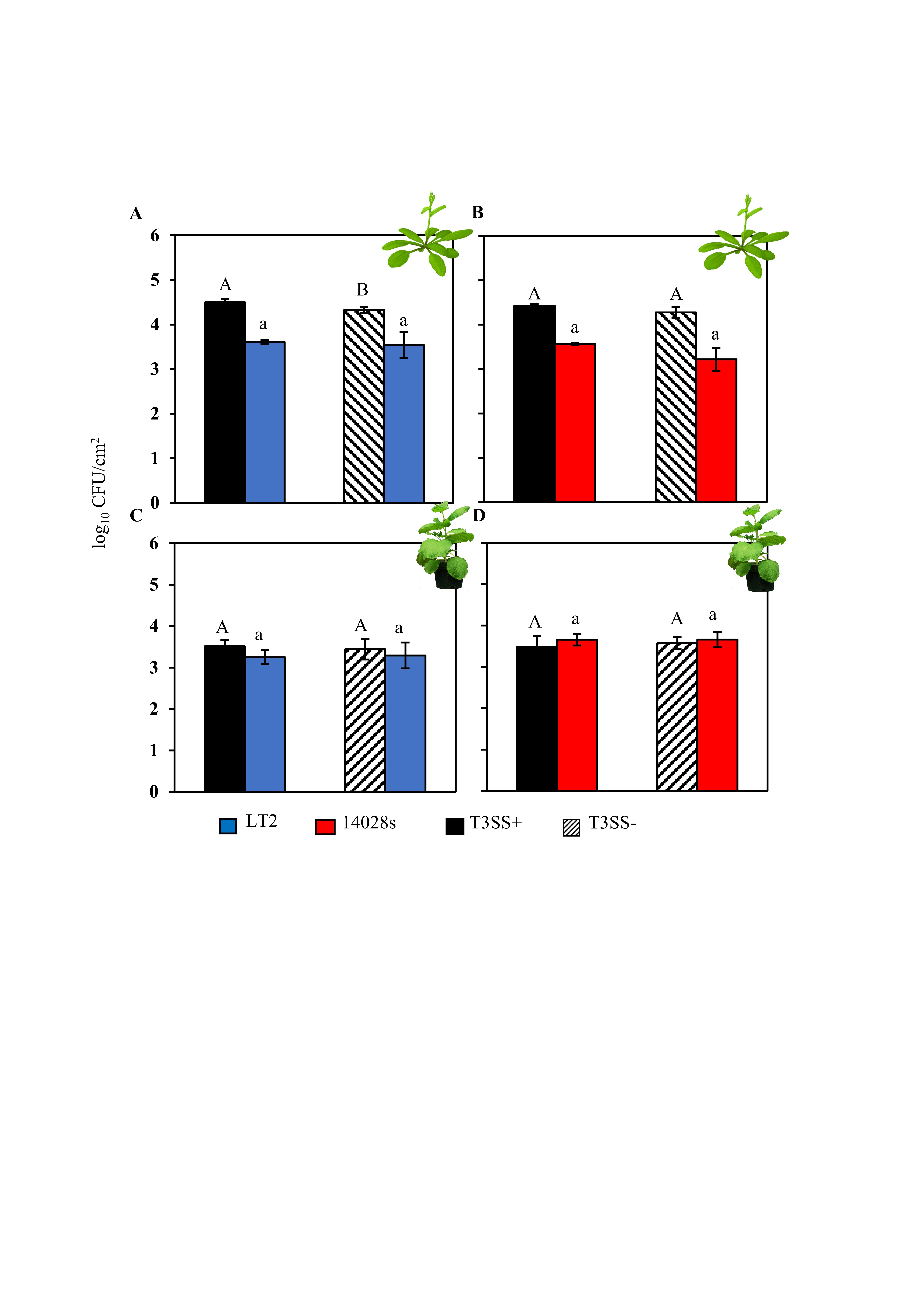

Supplement: SUPPLEMENTARY FIGURE S1 — Starting population of Salmonella enterica LT2, 14028s and the co-inoculated Pseudomonas syringae partner strains in Arabidopsis thaliana and Nicotiana benthamiana. Bacterial populations of S. enterica strains (A,C) 14028s (red), and (B,D) LT2 (blue) with co-inoculation partner P. syringae (Pto) or Pto∆hopQ with a functional Type III Secretion System (T3SS+, black) and Pto∆hrcC without a functional T3SS (T3SS−, striped). Inocula were syringe infiltrated into model plant hosts, (A,B) A. thaliana Col-0 at a concentration of 5 × 10^6^ CFU mL−1 for Pto strains and 5 × 10^5^ CFU mL−1 for S. enterica strains and (C,D) N. benthamiana at a concentration of 5 × 10^5^ CFU mL−11 for all strains. Bacterial populations were measured as log colony forming units per cm2 of leaf tissue (log10 CFU/cm2) immediately after the inoculation. Data are means ± SD (n = 3 plants). Different letters indicate significant differences (2-tailed t-test for each strain at p < 0.05). Three independent experiments were conducted with similar results. [file Image_1.jpg]

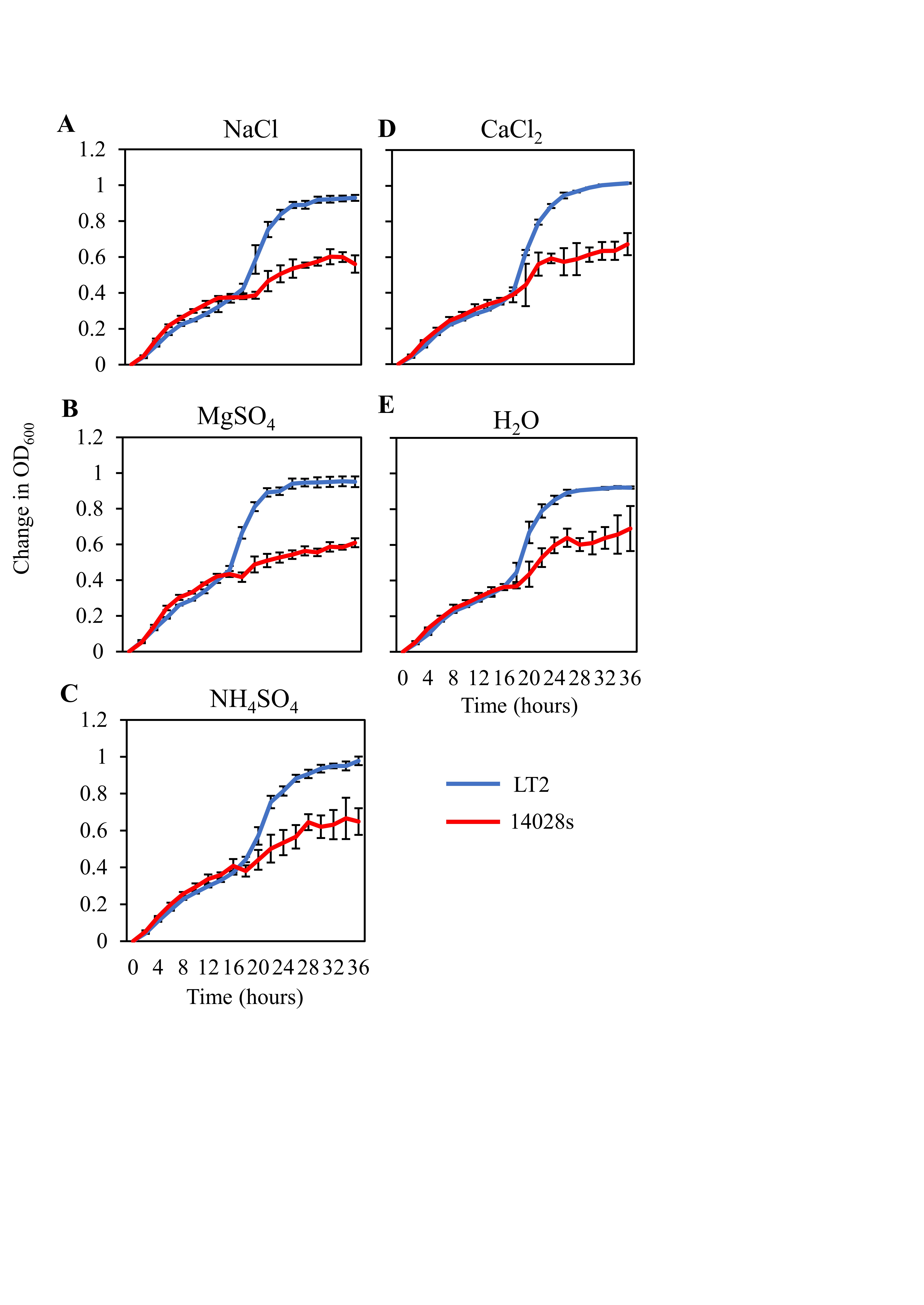

Supplement: SUPPLEMENTARY FIGURE S2 — Salmonella enterica biphasic growth in N. benthamiana apoplastic wash fluid is unaltered after augmentation with specific macro and micronutrients. Growth curves of S. enterica LT2 (blue) and S. enterica 14028S (red) in N. benthamiana apoplastic wash fluid augmented with (A) 10 mM sodium chloride, (B) 5 mM magnesium sulfate, (C) 10 mM ammonium sulfate, (D) 1 mM calcium chloride, and (E) water. Cultures were aliquoted into 5 replicate wells, incubated at 22°C, and the OD600 was recorded every 2 h. Growth was measured as the average change in measured OD600 with standard deviation error bars (n = 5 wells). Two independent experiments were conducted with similar results. [file Image_2.jpg]

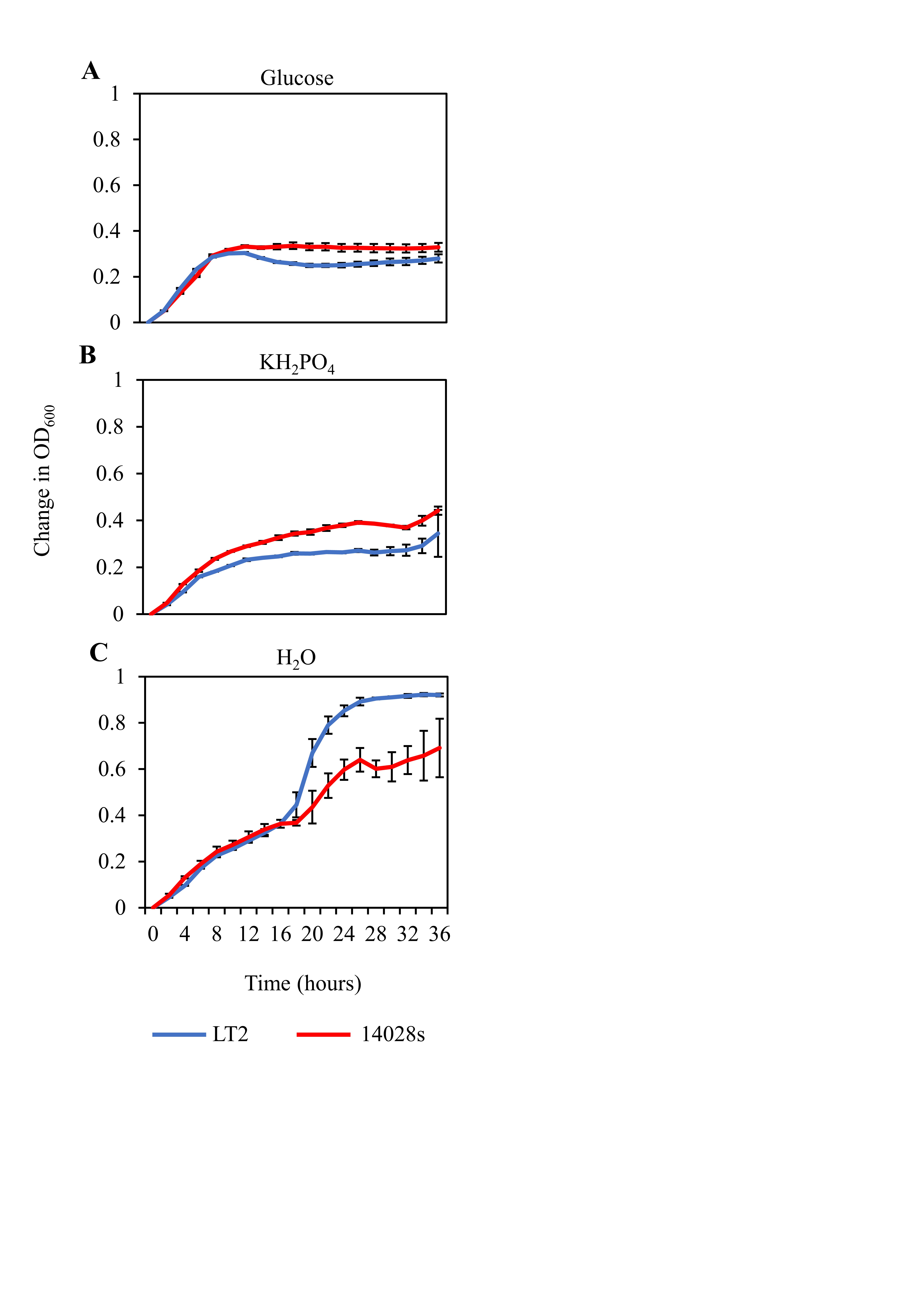

Supplement: SUPPLEMENTARY FIGURE S3 — Salmonella enterica strain biphasic growth in N. benthamiana apoplastic wash fluid is suppressed by glucose and phosphate augmentation. Growth curves of S. enterica LT2 (blue) and S. enterica 14028s (red) in N. benthamiana apoplastic wash fluid augmented with (A) 25 mM glucose, (B) 10 mM potassium phosphate, and (C) water. Cultures were aliquoted into 5 replicate wells, incubated at 22°C, and the OD600 was recorded every 2 h. Growth was measured as the average change in OD600. Error bars show standard deviation (n = 5 wells). Two independent experiments were conducted with similar results. [file Image_3.jpg]

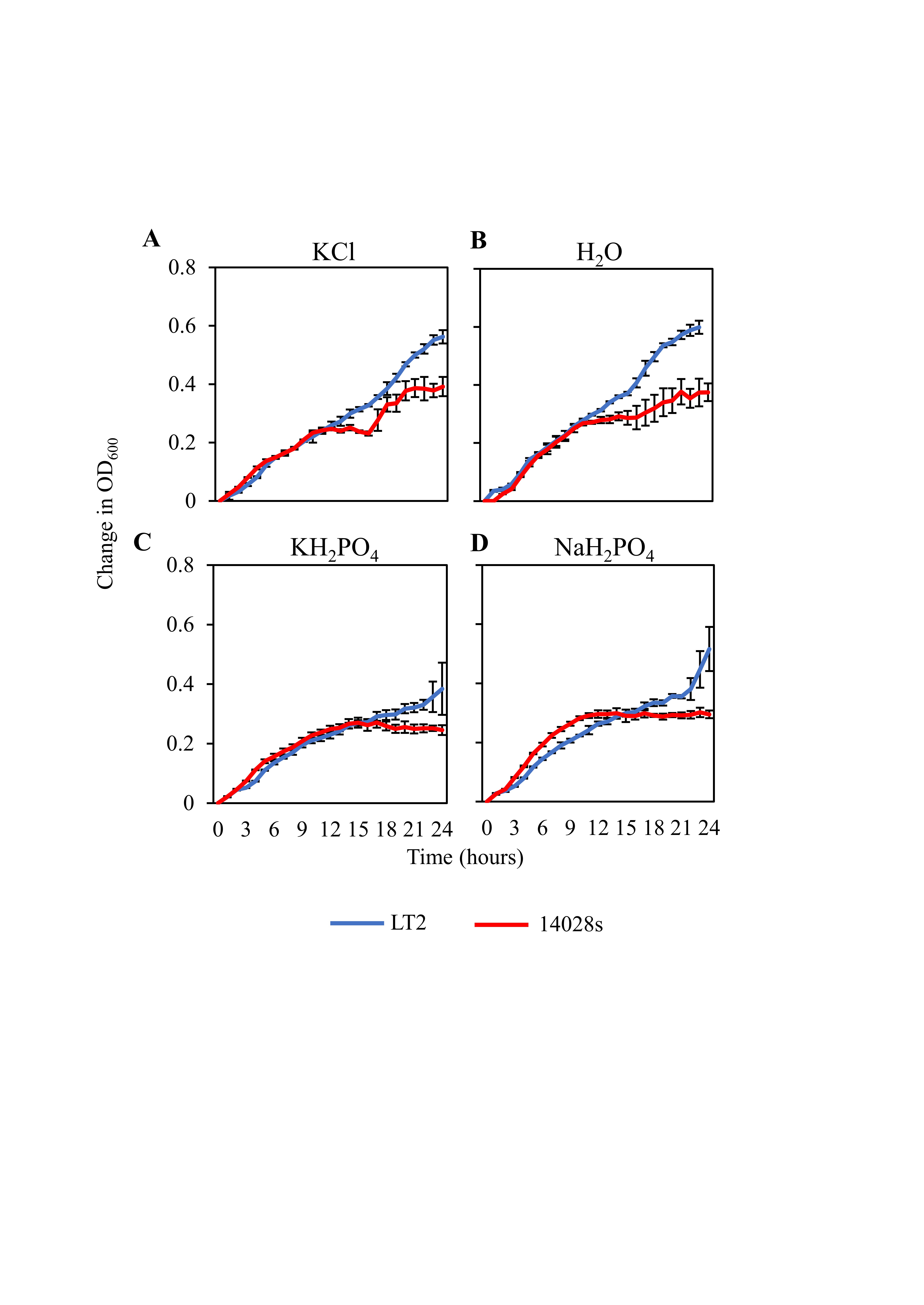

Supplement: SUPPLEMENTARY FIGURE S4 — Salmonella enterica biphasic growth in N. benthamiana apoplastic wash fluid is suppressed by the phosphate anion. Growth curves of S. enterica LT2 (blue) and S. enterica 14028s (red) in N. benthamiana apoplastic wash fluid supplemented with (A) 10 mM potassium chloride, (B) water, (C) 10 mM potassium phosphate, and (D) 10 mM sodium phosphate. Cultures were aliquoted into 5 replicate wells, incubated at 22°C, and the OD600 was recorded every hour. Growth was measured as the average change in measured OD600 with standard deviation error bars (n = 5 wells). Two independent experiments were conducted with similar results. [file Image_4.jpg]
