# Supplementary material for: RpoS contributes in a host-dependent manner to Salmonella colonization of the leaf apoplast during plant disease
Source: Front Microbiol. 2022 Nov 8;13:999183. doi: 10.3389/fmicb.2022.999183 (PMC9679226; doi:10.3389/fmicb.2022.999183)
Supplement: Supplementary file 5 [file Table_1.DOCX]

**Supplemental Table 1. Strains, plasmids, primers, and synthesized dsDNA used in this study**

| Strains | Relevant Characteristics | Source/ Reference | |
| --- | --- | --- | --- |
|  |  |  | |
| *Pseudomonas syringae* pv. *tomato* DC3000 (*Pto,* T3SS+) | Functional T3SS, compatible with *A. thaliana*, Rf^R^ | (1) | |
| *P. syringae* pv. tomato DC3000 ∆*hopQ1-1* (*Pto∆hopQ*, T3SS+) | Functional T3SS, deletion of *hopQ1-1* effector recognized by *N. benthamiana,* Rf^R^ | (2) | |
| *P. syringae* pv. tomato DC3000 ∆*hrcC* (*Pto∆hrcC*, T3SS-) | Mutation in outer membrane secretin protein of the T3SS, Rf^R^ | (3) | |
| *Salmonella enterica* serovar Typhimurium LT2 DM10000 (LT2) | *S. enterica* LT2 WT | Diana Downs collection | |
| *S. enterica* serovar Typhimurium 14028s (14028s) | *S. enterica* CDC 60-6516 derivative | Anna  Karls collection | |
| *S. enterica* serovar Typhimurium LT2 ∆*rpoS*1-352::rpoS_14028s_ (LT2 *rpoS*_14028s_ ) | Deletion of *rpoS* gene fragment and replaced with *rpoS* gene fragment from 14028s strain | This study | |
| Plasmids | Relevant Characteristics | Source/ reference | |
|  |  |  | |
| pR6KT2G | Gateway compatible R6K-based suicide vector for allelic exchange, *sacB*, Gm^R^, *gus,* Cm^R^ | (4) | |
| pR6KT2G:*rpoS*14028s | Suicide vector for complementation of *rpoS* gene fragment  *sacB*, Gm^R^, *gus* | This study | |
| pR6KT2G:*rpoS*del | Suicide vector for deletion of *rpoS* gene fragment  *sacB*, Gm^R^, *gus* | This study | |
| Synthesized oligonucleotides and dsDNA fragments | Nucleotide Sequences and Use | Source/ reference | |
|  |  |  | |
| pR6KT2G.F | 5’-GTCTTAAGCTCGGGCCCC -3’  pR6KT2G sequence confirmation | | (4) |
| pR6KT2G.R | 5’-GGGATATCAGCTGGATGGC -3’  pR6KT2G sequence confirmation | | (4) |
| PrpoS14028S.F | 5’-TTCTGCCCCGTATAGCCTG-3’  *rpoS* promoter sequence | | This study |
| rpoS14028S.F | 5’-CAAGGGATCACGGGTAGGAG-3’  *rpoS* sequence & PCR confirmation | | This study |
| rpoS14028S.inR | 5’-CCAGCAACGCCAGTCCAC-3’  *rpoS* sequence | | This study |
| rpoS14028S.outR | 5’-CAAGGGATCACGGGTAGGAG-3’  *rpoS* sequence & PCR confirmation | | This study |
| rpoS14028S | 5’-attB1-STM14_comp3086072 -3087023-attB2-3’  complement synthesized gene fragment | | This study  GenBank Accession: CP001363.1 |
| rpoSdelSpec | 5’-attB1-STM14_comp3086072-3086372-Spec- STM14_comp3086723-3087023-attB2-3’  deletion synthesized gene fragment | | This study  GenBank Accession: CP001363.1 |

References Cited

1. Buell CR, Joardar V, Lindeberg M, Selengut J, Paulsen IT, Gwinn ML, et al. The complete genome sequence of the Arabidopsis and tomato pathogen *Pseudomonas syringae* pv. *tomato* DC3000. Proc Natl Acad Sci USA. 2003;100(18):10181-6.

2. Wei CF, Kvitko BH, Shimizu R, Crabill E, Alfano JR, Lin NC, et al. A Pseudomonas syringae pv. tomato DC3000 mutant lacking the type III effector HopQ1-1 is able to cause disease in the model plant Nicotiana benthamiana. Plant J. 2007;51(1):32-46.

3. Penaloza-Vazquez A, Preston GM, Collmer A, Bender CL. Regulatory interactions between the Hrp type III protein secretion system and coronatine biosynthesis in Pseudomonas syringae pv. tomato DC3000. Microbiology (Reading). 2000;146 ( Pt 10):2447-56.

4. Stice SP, Thao KK, Khang CH, Baltrus DA, Dutta B, Kvitko BH. Thiosulfinate Tolerance Is a Virulence Strategy of an Atypical Bacterial Pathogen of Onion. Curr Biol. 2020;30(16):3130-40 e6.
